# A preliminary integrated genetic map distinguishes every chromosome pair and locates essential genes related to abiotic adaptation of *Crassostrea angulata*/*gigas*

**DOI:** 10.1186/s12863-018-0689-5

**Published:** 2018-11-15

**Authors:** Ismael Cross, Silvia Portela-Bens, Aglaya García-Angulo, Manuel A. Merlo, María E. Rodríguez, Thomas Liehr, Laureana Rebordinos

**Affiliations:** 1 Area de Genética. Facultad de Ciencias del Mar y Ambientales, Universidad de Cádiz. Polígono Río San Pedro, 11510 Puerto Real, Cádiz, Spain; 2 Institut für Humangenetik, Universitätsklinikum Jena, 07743 Jena, Germany

**Keywords:** *Crassostrea angulata*, *Crassostrea gigas*, Chromosome mapping, Abiotic adaptation, Aneuploidy, Chromosome markers

## Abstract

**Background:**

The re-sequencing of *C. angulata* has revealed many polymorphisms in candidate genes related to adaptation to abiotic stress that are not present in *C. gigas*; these genes, therefore, are probably related to the ability of this oyster to retain high concentrations of toxic heavy metals. There is, in addition, an unresolved controversy as to whether or not *C. angulata* and *C. gigas* are the same species or subspecies. Both oysters have 20 metacentric chromosomes of similar size that are morphologically indistinguishable. From a genomic perspective, as a result of the great variation and selection for heterozygotes in *C. gigas*, the assembly of its draft genome was difficult: it is fragmented in more than seven thousand scaffolds.

**Results:**

In this work sixty BAC sequences of *C. gigas* downloaded from NCBI were assembled in *BAC-contigs* and assigned to BACs that were used as probes for mFISH in *C. angulata* and *C. gigas*. In addition, probes of *H3*, *H4 histone*, *18S* and *5S rDNA* genes were also used. Hence we obtained markers identifying 8 out the 10 chromosomes constituting the karyotype. Chromosomes 1 and 9 can be distinguished morphologically. The bioinformatic analysis carried out with the BAC-contigs annotated 88 genes. As a result, genes associated with abiotic adaptation, such as metallothioneins, have been positioned in the genome. The gene ontology analysis has also shown many molecular functions related to metal ion binding, a phenomenon associated with detoxification processes that are characteristic in oysters. Hence the provisional integrated map obtained in this study is a useful complementary tool for the study of oyster genomes.

**Conclusions:**

In this study 8 out of 10 chromosome pairs of *Crassostrea angulata/gigas* were identified using BAC clones as probes. As a result all chromosomes can now be distinguished. Moreover, FISH showed that *H3* and *H4* co-localized in two pairs of chromosomes different that those previously escribed. 88 genes were annotated in the BAC-contigs most of them related with Molecular Functions of protein binding, related to the resistance of the species to abiotic stress. An integrated genetic map anchored to the genome has been obtained in which the BAC-contigs structure were not concordant with the gene structure of the *C. gigas* scaffolds displayed in the Genomicus database.

**Electronic supplementary material:**

The online version of this article (10.1186/s12863-018-0689-5) contains supplementary material, which is available to authorized users.

## Background

The worldwide production of oysters is 5.15 million tons/year, second only by weight to carp and other cyprinids [1]; the genera *Ostrea* and *Crassostrea* (Ostreidae) make up most of the species of commercial interest. The Pacific oyster *Crassostrea gigas* (Thunberg, 1793), in particular, is now one of the most important bivalves produced commercially throughout the world. Global annual production volume is uncertain, owing to taxonomic confusion in reports from China and other countries [2], but is conservatively estimated at 626,000 metric tons in 2016 [1]. Another *Crassostrea* species of interest is the Portuguese oyster, *C. angulata* (Lamarck, 1919); this species is an edible cupped oyster of major commercial importance and wide geographic distribution. It is assumed to be native to the Northwest Pacific region and has been introduced into many countries around the world [3]. *C. angulata* was a species of considerable commercial importance in Europe up to the early 1970’s, but serious mortalities in those years almost wiped out the Portuguese oyster from Europe. As a result, the extent of remaining *C. angulata* populations is poorly known. Recent studies have shown that the Fujian oyster that has been harvested and cultivated for more than 2000 years in coastal river mouths and estuaries of southern China is actually *C. angulata*. The production of this Fujian oyster accounts for about 50% of total oyster production in China. At the present time, according to some authors, *C. angulata* should be considered as a subspecies of *C. gigas* [3] . Because of the commercial importance of *Crassostrea* species, a number of breeding programs have been initiated over the years, and substantial genetic and genomic knowledge has been accumulated [4–9], although some important characteristics of genome organization remain unclear.

Oysters have a diploid number of 20 chromosomes [10]. Their karyotypes show most of their chromosomes to be submetacentric or metacentric, that are of similar size and morphologically indistinguishable; molecular markers are needed to differentiate them, mainly because of the many instances of aneuploidy occurring in natural populations of mollusks [11]. To study the evolution and phylogenetic relationships among oyster species, it is necessary to characterize the chromosomes individually, and particular regions of them. The C-banding and restriction techniques, as well as the study of Ag-NOR and cytogenetic markers, have been applied to several different species of oysters describing several cytogenetic markers that allow identify chromosome pairs in some cases [12–17].

The introduction of the fluorescence in situ hybridization (FISH) technique opened the possibility of studying more deeply inside the chromosomes than had been possible before. FISH is also a powerful tool for defining the cytogenetic location and relative order of DNA sequences, thereby anchoring the genome sequence to the chromosomes. It has been successfully used to integrate genetic and cytogenetic maps in many plants and animals [18–22]. During the past two decades, there have been many FISH studies, based on species of interest in aquaculture, that have contributed to a better understanding of these genomes. The FISH technique has greatly facilitated comparative genomic analysis to study the evolution of genomes [23–27]. In oysters, analysis has been focused on the study of repetitive and multigene families [28–31].

Histones are basic structural proteins of the nucleosomes and, in eukaryotic organisms, are essential for DNA packaging. This group is subdivided into five protein classes, termed H1, located bound to the “linker DNA” region between nucleosomes, and H2A, H2B, H3, and H4, located in the core of the nucleosomes. The histones have functional consequences, as *trans*-acting regulatory factors, because the transcription machinery must gain access to their specific binding sites at the DNA level [32]. Thus, knowing its location can help to understand its chromosome organization and evolution. In addition, the histone genes have been used as markers for taxonomic differentiation [33–36].

Going beyond the controversy of whether *C. angulata* and *C. gigas* are different species or subspecies, the whole genome re-sequencing of *C. angulata* showed many polymorphisms in candidate genes related to adaptation to abiotic stress that are not present in *C. gigas*, and hence are probably related to the ability of this oyster to retain high concentrations of toxic heavy metals [37].

Genetic maps are essential tools, useful for mapping quantitative-trait loci in marker-assisted selection, positional cloning, and genome assembly. In oyster species, linkage and physical maps have been described [38, 39]. Although the publication of the *C. gigas* genome draft is considered a great step forward in the knowledge of these organisms, at the present time the genome is assembled only to a level below that of the scaffold (N50 = 31,239) due to the complexity of its genome, its variation and selection for heterozygotes [40]; currently more than seven thousand scaffolds have been found. Mapping using BAC-FISH probes has allowed progress, in a straightforward and easy way, to final assembly and mapping of genes in their chromosomes. However, cytogenetic mapping of clones with large inserts is at present limited to a few species of interest for aquaculture [18, 23, 26, 27]. Knowing from gene libraries the position of clones that contain large inserts presents important advantages, since it is not limited to the location of repetitive DNA and allows the localization of multiple probes for each chromosome, thereby raising the number of markers per chromosome karyotype and in each species. From these clones, the inserts can be sequenced with next generation sequencing techniques, thereby enabling us to know the position and insert genes in the library hybridized in a straightforward manner [23, 26]. Moreover, the application of FISH based on the use of bacterial artificial chromosome clones (BAC) as probes provides an efficient approach for anchoring linkage and genomic data in the physical chromosomes [41]. In mollusks, genetic and cytogenetic maps have been integrated using this BAC-FISH approach in the Zhikong Scallop, *Chlamys farreri*, selecting markers from microsatellite-based linkage maps from a BAC library [18]. In oysters, to date, this approach has been applied only in the American oyster *C. virginica*, using inserts from a library based on phage P1 [39].

In order to gain insight into the genomes and chromosomes of *C. angulata/gigas,* an integrated genetic map, based on BACs and multigene families, has been designed with the aim of providing chromosomal markers and that could be anchored reliably to the sequenced genome of the species.

## Material and methods

### Selection and BAC clones getting from a *C. gigas* genetic library

Sixty BAC sequences downloaded from the National Center for Biotechnology Information (NCBI: http://www.ncbi.nlm.nih.gov/) (Acc. numbers from GU207404 to GU207462, and GU324325) were assembled with SeqMan software (*Lasergene, DNASTAR*). These sequences correspond to different BAC clones from a genetic library of *C. gigas* belonging to the Clemson University Genomics Institute (CUGI). From the contigs obtained (from now on named BAC-contigs), one BAC per contig and a single BAC (not included in any contig) were selected and ordered from the CUGI (Additional file 1: Table S1). The BAC clones were then mapped on *C. angulata* and *C. gigas* chromosomes by means of 2- and multi-color FISH.

### Probes and labelling for FISH

DNA was extracted from BACs using the BACMAX^tm^ DNA purification kit (Epicentre), following manufacturer’s instructions. For 2-color FISH, probes were labeled using the DIG/BIO Nick Translation Mix kit (Roche Molecular Biochemicals). In order to label 5S rDNA and 18S rDNA multigene families, PCR amplifications with biotin-16-dUTP or digoxigenin-11-dUTP (Roche Molecular Biochemicals) were made [28]. For the H3 histone gene the primers H3F (5’-CGTAAATCCACTGGAGGCAAGG-3′) and H3R (5’-GGATGGCGCACAGGTTGGTGTC-3′) were used [42]. The H4 histone genes were amplified by primers H4-F (5’-TGAGAGATAACATCCAGGGTATCAC-3’) and H4-R (5′- CTCTTGAGGGCGTAGACAACGTCCAT-3′).

### Chromosome preparations and FISH mapping

Samples of *C. angulata* of 2–3 cm in length were obtained from naturally-occurring populations located in the mouth of the River Guadalquivir, in Sanlúcar de Barrameda (Cádiz, Spain). *C. gigas* samples came from the facilities of a local aquaculture company. After feeding the oysters with phytoplankton for several days, they were placed in beakers and cultured for 8 h in colchicine. The gills were then extracted and put under hypotonic shock conditions by incubating with KCl 0.4% for at least 1 h. After that, they were transferred to fresh Carnoy for 1 h [28].

Twelve BACs were used as probes to hybridize on *C. angulata* and *C. gigas* chromosomes using the FISH-BAC technique (Table 1; Additional file 1: Table S1). In addition, probes of H3 and H4 histones, 18S and 5S rDNA genes were also used to analyze their position in chromosomes relative to the hybridized BACs.

**Table 1:** List of contigs after assembling BAC sequences of *Crassostrea gigas*

BACs – Acc. Numb. (GenBank)	BAC-hybrydyzed	BAC Size (bp)	Contig -Label	BAC-Contig Size (bp)
GU207429	**Ba106O6**	158.090	Contig-1	271.505
GU207449	Ba127B8	134.205		
GU207421	Ba21O17	164.533		
GU207416	Ba33M18	136.953		
GU207407	Ba3I17	172.567		
GU207433	Ba58D2	133.199		
GU207422	Ba67A11	203.422		
GU207441	Ba91N8	162.424		
GU207454	**Ba3D14**	176.912	Contig-2	254.895
GU207439	Ba30A15	137.420		
GU207409	**Ba133H22**	143.744	Contig-3	161.079
GU207424	Ba183L3	161.003		
GU207404	**Ba13P13**	152.407	Contig-4	222.359
GU207443	Ba181L20	124.416		
GU207447	Ba45N13	130.545		
GU207453	Ba98C1	143.273		
GU207446	Ba98I23	114.800		
GU207426	**Ba17L12**	119.765	Contig-5	204.747
GU207445	Ba172M23	130.127		
GU207458	Ba118P9	174.057		
GU207462	Ba80D21	123.070		
GU207460	**Ba96P24**	132.147	Contig-6	141.754
GU207420	Ba171O9	123.180		
GU207448	Ba107F17	123.528		
GU207455	**Ba45B21**	138.330	Contig-7	237.501
GU207406	Ba147A6	132.499		
GU207436	Ba96I10	126.652		
GU207411	**Ba18I16**	164.184	Contig-8	238.060
GU207450	Ba8F15	117.743		
GU207413	**Ba102L1**	122.190	Contig-9	198.436
GU207442	Ba151B13	139.968		
GU207423	Ba50J23	84.264		
GU207459	**Ba177C12**	136.235	Contig-10	195.233
GU207456	Ba188K9	117.224		
GU207412	**Ba50F9**	130.402	Contig-11	277.695
GU324325	Ba57G5	149.919		
GU207418	**Ba127F5**	132.710	Contig-12	132.710

BACs in bold were hybridized by means of 2-color and multiple-color BAC-FISH technique

The 2-color FISH method used for BAC clones probes was performed following the protocol of García-Cegarra et al. [26]. Multiple-FISH (mFISH) was carried out as described in Portela-Bens et al. [23]. The 2-color FISH technique described by Cross et al. [28] was used to localize the H3 and H4 histone genes.

### Bioinformatic analysis of BAC clones

The contigs containing hybridized BACs were annotated from the *C. gigas* genome draft, available at the NCBI [40]. The BAC-contig sequences were subjected to Blast search against *C. gigas* Whole-Genome shotgun contigs from NCBI/BLAST/blastn suite (from now on named Cg-contigs) (AFTI: *Crassostrea gigas* strain 05 × 7-T-G4–1.051#20, whole genome shotgun sequencing project). In order to obtain genes from these Cg-contigs, a manual search of scaffolds (containing Cg-contigs) stored in the “EnsemblMetazoa *C. gigas”* database was conducted. To confirm the presence of this set of genes obtained after scaffolds data mining, the genes were then blasted (Blast2seqs) against every single BAC-contig; genes showing a *P*-value < 0.001 and a coverage > 90% were then annotated. Next, using the genomic editor Apollo [43], and Geneious basic 5.6.5 (http://www.geneious.com), the results were individually tested and adjusted in the final editing process.

The study of functions in genome regions covered by the BAC-contigs was carried out using data mining techniques based on the information stored in the Gene Ontology (GO) database. After getting GO terms from genes annotated in the BAC-contigs (*C. gigas* EnsemblMetazoa) we have used the QuickGo browser (https://www.ebi.ac.uk/QuickGO) to obtain GO terms found for each BAC-contig, and then analyzed the main Molecular Function carried out in those regions. Comparison charts from the Biological Process, Molecular Function and Cellular Component domains were also prepared.

Finally, the structure of the BAC-contigs, obtained after annotation, and the information provided by the Genomicus database v25.01 using *C. gigas* as reference species [44], were compared. The object of this analysis is to test the concordance between, on the one hand, the *C. gigas* genome assembly and structure, available in *C. gigas* databases (EnsemblMetazoa *C. gigas* and Genomicus), and on the other, the BAC-contigs sequences. To carry out this analysis, a search was made for every gene annotated finally in BAC-contigs in the Genomicus database (using *C. gigas* genome as reference), and the scaffolds containing the annotated genes were scored. Then, for each BAC-contig, a map of scaffolds containing the genes was created. This information was used to compare the BAC-contig genetic structure obtained after annotation *vs* the *C. gigas* scaffolds structure from *C. gigas* genomic databases.

## Results

### BAC-FISH

Sixty BAC sequences of *C. gigas* were downloaded from NCBI and assembled in BAC-contigs by means of Seq-Man software *(Lasergene, DNASTAR)*. Eleven BACs from these contigs and one BAC more, not belonging to contigs (BAC-unitig), were used to carry out this study.

**Fig. 1:**
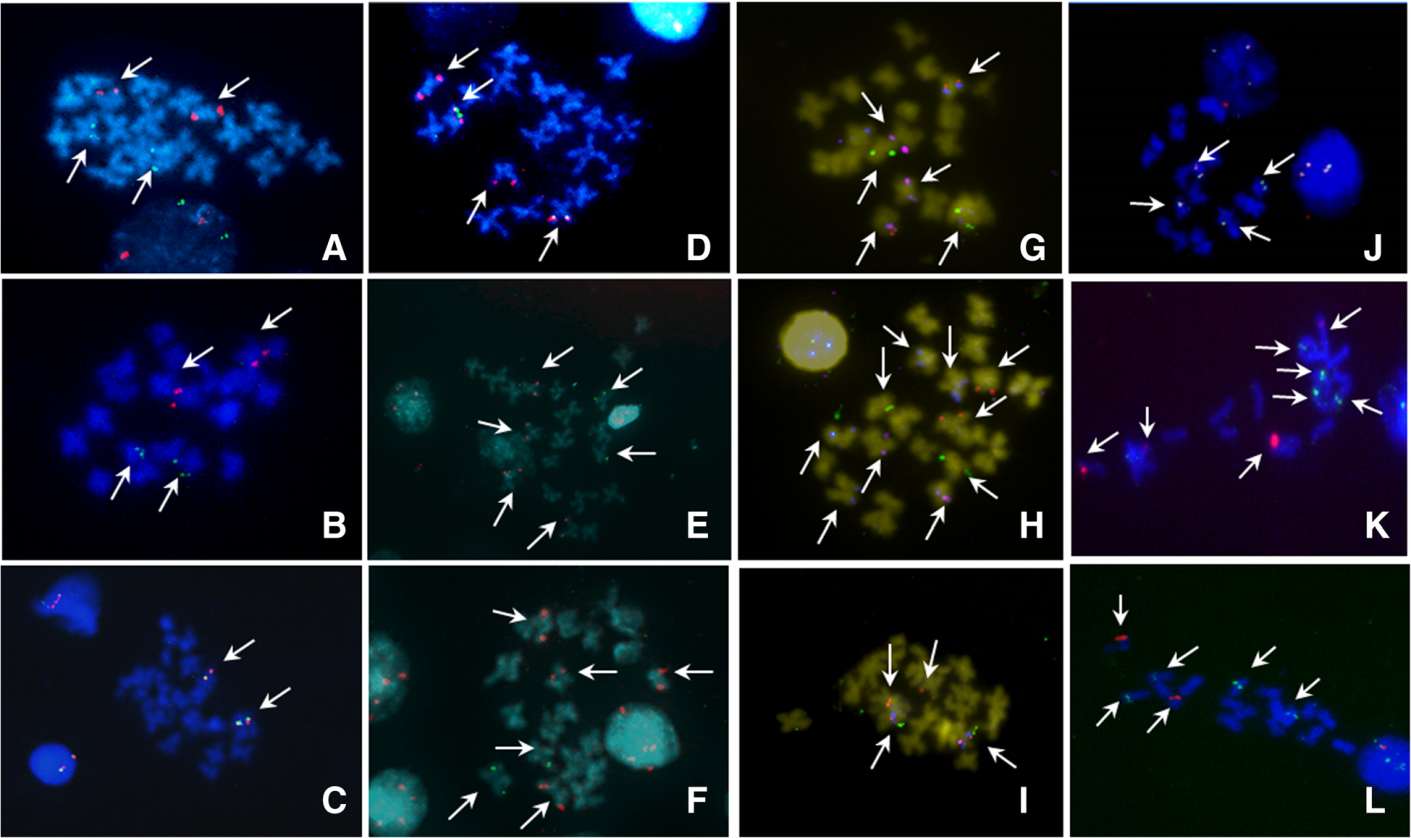
mFISH of the BACs isolated from the library that contained the following probes (genes and BAC-clones): **a**) 3D14 (red), *18S rDNA* (green), **b**) 127f5 (red) 18i16 (green), **c**) 18I16 (red), 17 L12 (green), **d**) 3D14 (red), *5S rDNA* (green), **e**) 127f5(red), *5S rDNA* (green), **f**) 18I16 (red), *5S rDNA* (green), **g**) 17 L12 (green), 45B21 (orange), 96P24 (pink), 13P13 (blue), **h**) 133H22 (green), 127F5 (orange), 18I16 (pink), *5S rDNA* (blue), **i**) 177C12 (green), 127F5 (orange), 50F9 (pink), 102 L1 (blue), **j**) *H3 histone* (green), *H4 histone* (red), **k**) *H3 histone* (green), *5S rDNA* (red), **l**) *H4 histone* (green), *18S rDNA* (red). Pictures A, D, E, F and H correpond to *C. angulata* metaphasic plates. The remaining plates correspond to *C. gigas* species

**Fig. 2:**
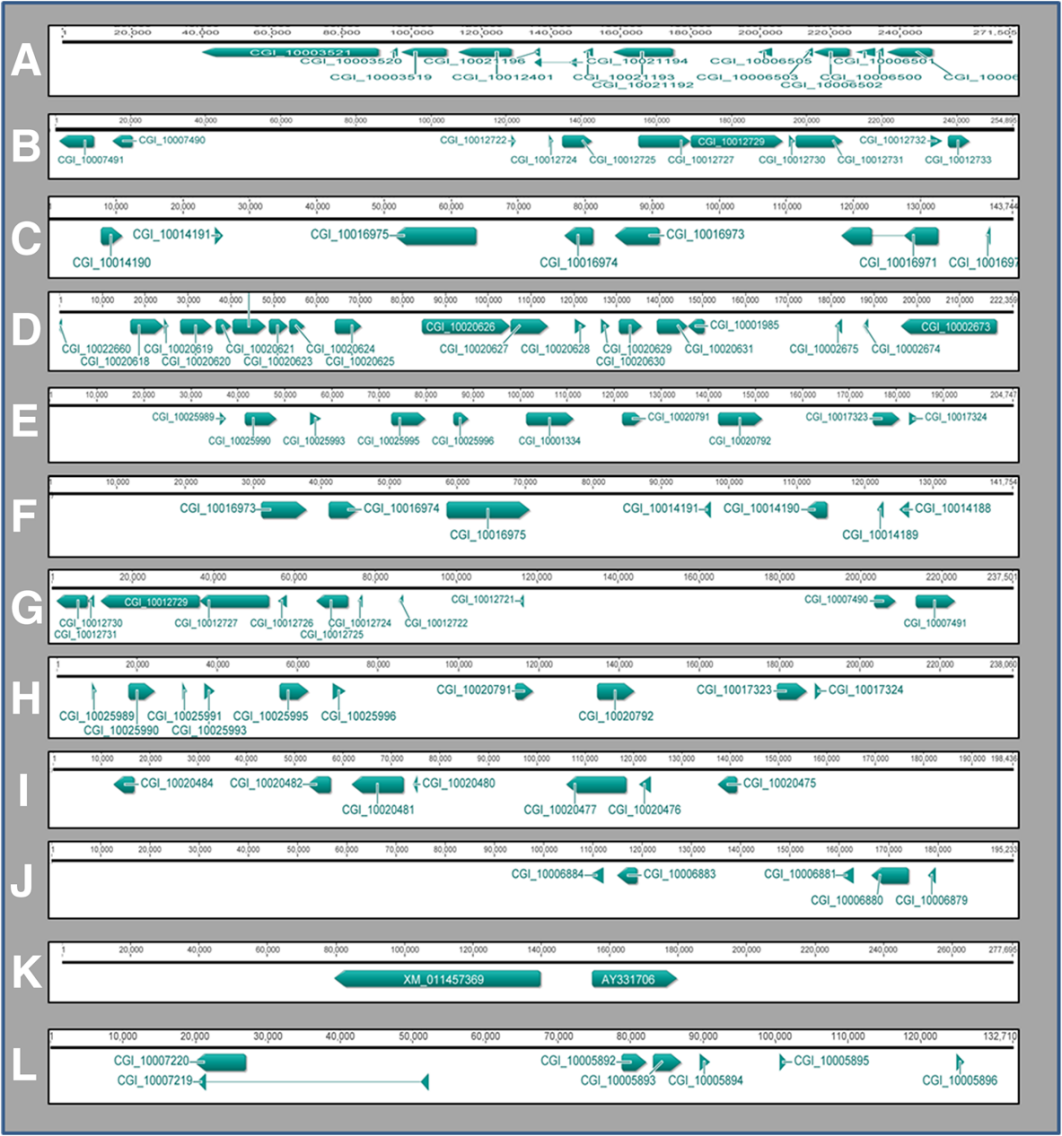
Annotation of *C. gigas* BAC-contigs. Genes are indicated by arrows and are labeled with the ID of genes as named in the *C. gigas* genome database. A-K correspond to BAC-contigs 1-11 respectively. L corresponds to BAC127F5

No differences in BAC-FISH results between *C. angulata* and *C. gigas* were found during the study. Results show three BACs hybridized in chromosome 2: 177C21, 50F9 and 102 L1 (Fig. 1). The last two are located in one arm of the chromosome. BAC 177C21 is located in the other arm, in subcentromeric position. Chromosome 3 contains the H4 histone gene and, in the same arm, the clones Ba17L12 and Ba18I16 are found co-localized. BAC Ba106O6 is located in chromosome 4 next to the 5S rDNA genes. The 5S rDNA genes are located in two chromosome pairs, 4 and 5, in *C. angulata* and *C. gigas* (Cross et al. 2005; Benabdelmouna et al. 2008); this BAC is located in a pericentromeric position, in a more internal position than 5S rDNA. The BAC 3D14 is found in chromosome 5, next to another 5S rDNA locus. Following these results, these two BACs allow chromosome 4 to be distinguished from chromosome 5 in these *Crassostrea* oysters studied (Table 2; Figs. 1 and 2). BAC 127F5 is located in a pericentromeric position of chromosome pair 6. BACs 133H22 and 96P24 are located in chromosome pair 7; and the BACs 13P13 and 45B21 are in chromosome 8. Of the 12 BACs hybridized, none is located in chromosomes 1 or 9. The 18S rDNA gene family is located in chromosome 10 [28].

**Table 2:** Summary of results obtained after hybridization of BACs by two- and multi-color FISH technique and annotation of BAC-contigs and a BAC-unitig

Chrom.	Probe	BAC- contig	Genes
**1**	--	**–**	--
**2**	**BAC Ba 102 L1**	Contig 9	*NF-kappa-B inhibitor cactus; DNA replication complex GINS protein PSF1; Adenylosuccinate synthetase; Mitochondrial import inner membrane translocase subunit Tim9; Novel protein coding; Enhanced at puberty protein 1-like protein B; Microtubule-associated proteins 1A/1B light chain 3C.*
	**Ba177C21**	Contig 10	*Novel protein coding; ADAMTS-like protein 3; F-box only protein 22; Novel protein coding; Novel protein coding;*
	**Ba50F9**	Contig 11	*MT-IIG (C. virginica), Filamin-A-like (C.gigas predicted)*
**3**	**H4**	--	H4 histone
	**BAC Ba17L12**	Contig 5	*Ribonuclease P protein subunit p40; Kelch-like protein 13; Sodium-and chloride-dependent betaine transporter; Pleckstrin-like protein domain-containing family B member 2; Proteasome subunit beta type-8; Novel protein coding; Novel protein coding; Steroid 17-alpha-hydroxylase/17,20 lyase; E3 ubiquitin-protein ligase MYLIP; Pyruvate dehydrogenase phosphatase regulatory subunit, mitochondrial*
	**BAC Ba18I16**	Contig 8	*Kelch-like protein 13; Sodium-and chloride-dependent betaine transporter; Pleckstrin-like protein domain-containing family B member 2; Proteasome subunit beta type-8; Novel protein coding; Novel protein coding; Novel protein coding; Steroid 17-alpha-hydroxylase/17,20 lyase; E3 ubiquitin-protein ligase MYLIP; Pyruvate dehydrogenase phosphatase regulatory subunit, mitochondrial;*
**4**	**5S rDNA**	--	5S Ribosomal DNA
	**BAC Ba106O6**	Contig 1	*Metallothionein; Novel protein coding; Filamin-C; Novel protein coding; Novel protein coding; Nudix hydrolase 20; JmjC domain-containing protein C2orf60-like protein; Novel protein coding; Novel protein coding; Tripartite motif-containing protein 3; Neurogenic locus notch-like protein 4; Caveolin; Metallothionein; Metallothionein;*
**5**	**5S rDNA**	--	*5S ribosomal DNA*
	**BAC Ba3D14**	Contig 2	*Myeloid differentiation primary response protein MyD88; Novel protein coding; Myeloid differentiation primary response protein MyD88; Novel protein coding; Myeloid differentiation primary response protein MyD88; Putative GTP-binding protein 6; LIM domain and actin-binding protein 1; Novel protein coding; Novel protein coding; Calcineurin-binding protein cabin-1; Di-N-acetylchitobiase.*
**6**	**Ba127F5**	Unitig-Ba127F5	*Serine/threonine-protein kinase 19; Protein Dom3Z; ZZ-type zinc finger-containing protein 3; Rhodopsin, GQ-coupled; C3a anaphylatoxin chemotactic receptor; Melatonin receptor type 1B; Melatonin receptor type 1B; G-protein coupled receptor moody.*
**7**	**BAC Ba133H22**	Contig 3	*Thyroid transcription factor 1-associated protein 26; Orexin receptor type 2; Novel protein coding; WD repeat-containing protein 86; WD repeat-containing protein 86; Novel protein coding; Novel protein coding; Cys-loop ligand-gated ionic channel.*
	**BAC Ba96P24**	Contig 6	*Galanin receptor type 2; Alpha-2A adrenergic receptor; Thyroid transcription factor 1-associated protein 26; Orexin receptor type 2; Novel protein coding; Novel protein coding; Cys-loop ligand-gated ionic channel.*
**8**	**BAC Ba13P13**	Contig 4	*Novel protein coding; Tripartite motif-containing protein 45; Complement C1q-like protein 3; Tripartite motif-containing protein 45; NADH dehydrogenase [ubiquinone] 1 beta subcomplex subunit 11, mitochondrial; Novel protein coding; Neurogenic locus notch-like protein 2; Putative imidazolonepropionase; Coiled-coil domain-containing protein 37; Ribonuclease P protein subunit p30; Novel protein coding; Tryptase gamma; Protein sidekick-2; Tyrosine-protein phosphatase 99A; Metalloproteinase inhibitor 1; Tissue inhibitor of metalloproteinase TIMP; Metalloproteinase inhibitor 3; Tissue inhibitor of metalloproteinase TIMP 1.1; WD40 repeat-containing protein SMU1; N-acetyltransferase ats1; Novel protein coding.*
	**BAC Ba45B21**	Contig 7	*Myeloid differentiation primary response protein MyD88; Novel protein coding; Heavy metal-binding protein HIP; Myeloid differentiation primary response protein MyD88; Novel protein coding; Myeloid differentiation primary response protein MyD88; Thioredoxin-like protein 4A; Putative GTP-binding protein 6; LIM domain and actin-binding protein 1; Novel protein coding; Novel protein coding; Disrupted in renal carcinoma protein 2-like protein*
**9**	--	--	--
**10**	**18S rDNA**	--	*18S ribosomal DNA*

BACs in bold were hybridized by means of 2-color and multiple-color BAC-FISH technique

H3, H4 and ribosomal genes (5S and 18S) probes were also hybridized by standard FISH protocol. The results showed that H3 and H4 co-localized in two pairs of chromosomes of medium size. One of them should be chromosome pair 3, as described in the preceding paragraph, by means of FISH-BAC protocols. The additional signal of hybridization was observed in a second chromosome pair of medium-size. Analysis of the relative position of histone genes and ribosomal genes showed that they are not co-localized in either the Japanese or Portuguese oysters (Figs. 1 and 2).

### Annotation and integrated genetic map

**Fig. 3:**
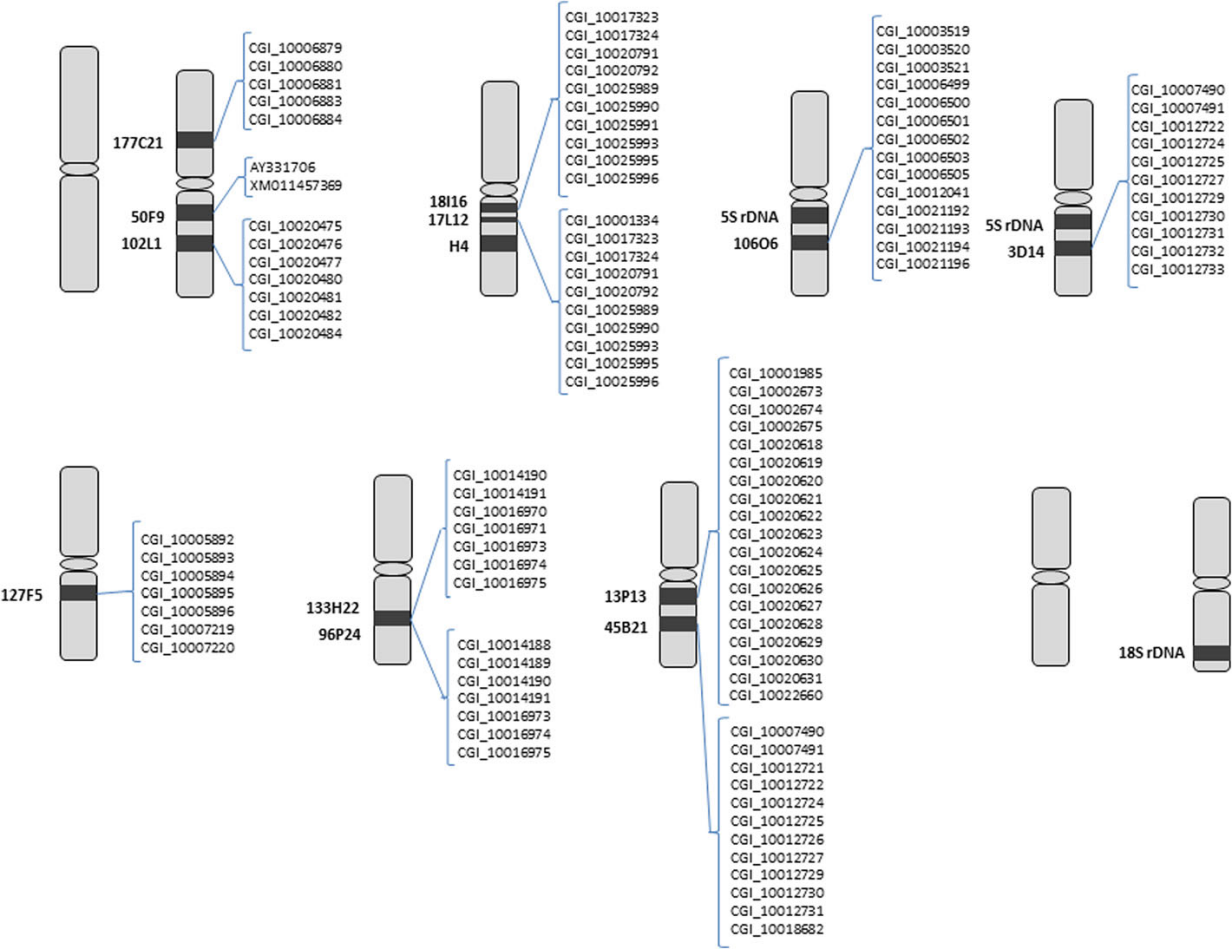
Integration of physical and cytogenetic maps of *Crassostrea angulata/gigas*. Cytogenetic results are shown in black boxes within the chromosome diagram. Labels indicate the ID of genes as named in the *C. gigas* genome database

Following the bioinformatic analysis carried out to annotate BAC-contigs, 88 genes were annotated in the BAC-contigs and mapped in *Crassostrea* chromosomes. In Table 2, Figure 2 and Additional file 2: Table S2, all the information about gene annotation is shown. Figure 3 shows the integrated map obtained after BAC-FISH and annotation analysis.

Results show that contig-1 arises from the assembly of eight BACs and it is 271,505 bp long (Table 1; Additional file 1: Table S1). This contig-1 is located in chromosome 4 and presents 14 genes, including 3 metallothionein genes (*MT*) and 5 *novel protein codings,* which are predicted genes described in the *C. gigas* Ensemble Metazoa database as being of unknown function.

Contig-2 is located in chromosome 5; it is 254,895 bp long (Table 1; Additional file 1: Table S1) and it contains 11 genes, four of them with unknown function. Some of the genes are associated with the myeloid differentiation primary response protein. Genes for *calcineurin-binding protein cabin-1* and *acetylchitobiose* are also present.

Contig-3 results from the assembly of two BACs: Ba133H22 and Ba183L3. The former was used as probe in the FISH analysis. Since this contig presented a wide region of non-homologies between the two BACs, the annotation was made mainly based on the BAC hybridized. After annotations, it can be seen that contig-3 includes 7 genes including a thyroid transcription factor and an orexin receptor. Three genes with unknown function are also found.

Contig-4 emerges from the assembly of 5 BAC sequences and has a length of 222,359 bp (Table 1; Additional file 1: Table S1). From the five BACs included in this contig, the BAC named Ba13P13 was used as a probe. Contig-4 includes the largest number of genes of all contigs analyzed. It contains 19 genes; four of them are novel protein genes. The 15 genes with known function include *metalloproteinase inhibitor 1, tissue inhibitor of metalloproteinase (TIMP)* and *acetyltransferase* (*ats1*), among others. Contig 4 is located in chromosome 8.

Contig-5 arises from the assembly of four BACs and it is 204,747 bp long (Table 1; Additional file 1: Table S1). To locate the contig in chromosomes of the two oyster species, BAC Ba17L12 was used as a probe. It localizes in chromosome 3 (Figs. 1 and 2). This contig has annotated 10 genes including *ubiquitin-protein ligase MYLIP, pyruvate dehydrogenase phosphatase regulatory subunit*, and *sodium and chloride-dependent betaine transporter,* among others. Two genes of unknown function were also annotated (Table 2; Additional file 1: Tables S1 and Additional file 2 Table S2).

Contig-6 includes 3 BAC sequences and it is 141,754 bp long (Table 1; Additional file 1 Table S1). BAC Ba96P24 was used as a probe to localize the contig and it hybridized in chromosome 7. The annotations of this contig showed that it contains 7 genes, five of them with known functions. Several of the genes, such as *thyroid transcription factor, orexin receptor type 2* and *cys-loop ligand-gated ionic channel genes*, are also present in contig 3, but others like *galanin receptor type 2* and *alpha-2A adrenergic receptor*, are not. Contig-3 is also present in chromosome 7 and co-localizes with this contig-6.

Contig-7 is 237,501 bp long (Table 1; Additional file 1: Table S1) and it emerges from 3 BACs sequences. It includes 12 genes, eight of them with functions including heavy metal-binding protein, *thioredoxin-like protein 4A* and *actin-binding protein 1*. Like contig-4, it localizes in chromosome 8.

Contig-8 comes from the assembly of two BAC sequences, Ba18I16 and Ba8F15, and it is 238,060 bp long (Table 1; Additional file 1: Table S1). The Ba18I16 BAC was used as a probe to localize it in chromosomes of the oysters *C. angulata* and *C. gigas*. Contig-8 co-localizes with contig-5 in chromosome 3. Genes annotated are the same in both contigs, except for *ribonuclease P protein subunit p40*, which is absent in contig-8.

Chromosome 2 showed a hybridization signal with 3 BACs, corresponding to three contigs: contig-9, 10 and 11. Contig-9 results from the assembly of 3 BAC sequences, and is 198,436 bp long (Table 1; Additional file 1: Table S1). The annotation of contig-9 showed 6 genes with known function and 1 novel protein coding. Among the six genes can be found *DNA replication complex GINS protein PSF1, adenylosuccinate synthetase*, and *microtubule-associated proteins 1A/1B light chain 3C.* Contig-10 is 195,233 bp long and arises from two BACs (Table 1; Additional file 1: Table S1). It has 5 genes, but only two of them (*ADMAMTS-like protein* and *F-box protein 22*) with known function. Contig-11 did not show a match with any genes annotated in the NCBI *C. gigas* database, so it was BLAST-searched against the nucleotide collection (nr/nt) and filtered for mollusks in the NCBI database. Two genes were found: *metallothionein* (*C. virginica* IIG) and *filamin-A-like* (*C. gigas* predicted).

The unitig BAC Ba127F5 is 132,710 bp long and it has 6 genes and one novel protein coding (Tables 1 and 2; Additional file 1: Table S1). Included in this BAC are the *galanin receptor type 2, orexin receptor type 2,* and *cys-loop ligand-gated ionic channel* genes.

The GO analysis showed that most genes in BAC-contigs showed Molecular Functions (GO term category) related to protein binding. In particular, we have observed an enrichment in the GO terms “metal ion binding” and “zinc ion binding” in most BAC-contigs: 1, 2, 5, 7–11 and unitig BAC 127F5 (Table 3; Additional file 3: Table S3; Additional file 4: Figure S1). Other functions include “hydrolase activity” (BAC-contigs 2 and 4), “GTP binding” (2, 7 and 9) or “G-protein coupled receptor activity” (BAC-contigs 3, 6 and BAC-unitig) were also enriched (Table 3).

**Table 3:** Summary of the top 4 Go Term per contig (Molecular Function Category)

Contig	Gene onthology term	Go term description
Contig 1	GO:0005515, GO:0000049, GO:0016706, GO:0046872	Protein binding, tRNA binding, oxidoreductase activity, metal ion binding.
Contig 2	GO:0005515, GO:0004553, GO:0005525, GO:0008270	Protein binding, hydrolase activity, GTP binding, zinc ion binding.
Contig 3	GO:0004930, GO:0004983, GO:0005230, GO:0005515	G-protein coupled receptor activity, neuropeptide Y receptor activity, extracellular ligand-gated ion channel activity, protein binding.
Contig 4	GO:0005515, GO:0016787, GO:0016810, GO:0016812	Protein binding, hydrolase activity, acting on carbon-nitrogen (but not peptide) bonds, hydrolase activity, acting on carbon-nitrogen (but not peptide) bonds, in cyclic amides.
Contig 5	GO:0005515, GO:0008270, GO:0004175, GO:0004298	Protein binding, zinc ion binding, endopeptidase activity, threonine-type endopeptidase activity.
Contig 6	GO:0004930, GO:0004983, GO:0005230	G-protein coupled receptor activity, neuropeptide Y receptor activity, extracellular ligand-gated ion channel activity.
Contig 7	GO:0005515, GO:0005525, GO:0008270	Protein binding, GTP binding, zinc ion binding.
Contig 8	GO:0005515, GO:0008270, GO:0004175, GO:0004298	Protein binding, zinc ion binding, endopeptidase activity, threonine-type endopeptidase activity.
Contig 9	GO:0000287, GO:0004019, GO:0005525, GO:0046872	Magnesium ion binding, adenylosuccinate synthase activity, GTP binding, metal ion binding.
Contig 10	GO:0005515, GO:0008233, GO:0008237, GO:0008270	Protein binding, peptidase activity, metallopeptidase activity, zinc ion binding.
Contig 11	GO:0051015, GO:0005515, GO:0046872	
BAC 127F5	GO:0004930, GO:0003677, GO:0008270, GO:0016301	G-protein coupled receptor activity, DNA binding, zinc ion binding, kinase activity.

### Genomic structure of BAC-contigs

The structure of the BAC-contigs, obtained after gene annotations, and the information about *C. gigas* gene order obtained from the Genomicus database, were compared. After testing concordance between the BAC-contigs structure and the scaffold assembly available in the *C. gigas* genome database (Table 4; Additional file 5: Figure S2), it was observed that most of the BAC-contigs structure were not concordant with the gene structure of the *C. gigas* scaffolds displayed in the Genomicus database. BAC-contig 1 presented 15 genes belonging partially to 4 scaffolds displayed in the Genomicus database, and they were incompatible with each other (Table 3; Additional file 4: Figure S1). BAC-contig 2 contains 11 genes and their relative position and genetic structure were not comparable to what is observed in the scaffolds of the Genomicus databases, where genes were found in partial regions of scaffold 870 and scaffold 617. BAC-contigs 3, 4, 5, 7, 8 and BAC 127F5 also presented scaffold incompatibility, being contained in a range of 2–4 incompatible scaffolds. Only in scaffolds 6, 9 and 10 could be observed a genetic structure compatible with scaffolds from the *C. gigas* genome databases, because genes annotated were present in single or several compatible scaffolds.

**Table 4:** Comparative analysis between genes annotated in BAC-contigs and scaffolds, from *C. gigas* genome database, where they would be contained

*BAC-Contig*	Number of genes	Number of scaffolds (*C. gigas* genome database)	Scaffold ID
1	15	4	Scaffold1297, scaffold852, scaffold99, scaffold1121.
2	11	2	Scaffold870, scaffold617
3	7	2	Scaffold631, scaffold117
4	19	4	Scaffold1360, scaffold410, scaffold244, scaffold38210
5	10	4	Scaffold425, scaffold34732, scaffold1004, scaffold 1900
6	7	2	Scaffold117, scaffold631
7	12	3	Scaffold617, scaffold189, scaffold870
8	10	3	Scaffold425, scaffold1004, scaffold1900
9	7	1	Scaffold388
10	5	1	Scaffold2448
11	2	–	ND
BAC127F5	7	2	Scaffold42558, scaffold41988

## Discussion

FISH has been proved to be an efficient method for correlating genetic and cytogenetic maps using marker-anchored BAC clones [18].

In the cytogenetic map of *C. angulata* and *C. gigas* oysters, following our results, we were able to identify 8 out of 10 chromosome pairs using BAC clones as probes. These results are relevant because the karyotype of the *Crassostrea* species presents a small range of morphology and sizes [10, 28], and it has not been possible to identify every one. In the oysters *C. angulata* and *C. gigas*, 5S rDNA genes are located in chromosomes 4 and 5 [28, 45] and the 5.8–18-28S rDNA genes in chromosome 10 [15, 46]. Our results show that chromosomes 4 and 5 can be identified by the position of Ba106O6 and Ba3D14 BAC clones. In relation to the H4 histone gene, when using BAC-FISH conditions, this gene was observed in chromosome pair 3. However, after using standard FISH protocols that are usually applied to multigene families [28], a second signal was detected in a medium-size chromosome. In *C. gigas* the location of the H3 gene has been described [42], but the position described is not consistent with our findings, since those authors described two hybridization signals on chromosome pairs 4 and 10. It could be argued that the position 3 observed in our results is different from chromosome pair 4, owing to an absence of chromosomal markers in that work leading to a mistake being made between the two chromosome pairs (3 and 4) that are almost identical in size and morphology. Our results show that the position is not in chromosome 4 as described by Boully et al. [42], because that position is occupied by 5S rDNA genes [28, 45]. However an even more relevant result is that histone genes are not in chromosome 10 [42], because that is the chromosome that bears major ribosomal genes described in the Pacific and Portuguese oysters [15, 46], and the double FISH carried out in this study confirms that the two families are not co-localized. For that reason, the location of the histone genes should be revised because contradictory results have been obtained in those experiments.

The GO term analysis showed an over-representation in BAC-contigs of molecular function associated with metal ion binding and zinc ion binding. In previous studies, enrichment was found in genes coding for proteins implicated in the detoxification of metal ions in polluted areas, in *C. angulata*, in comparison with those of *C. gigas* [37]. In this work we have localized a *metallothionein* gene in chromosome 2 and 4, in the BAC-contig 1. Metallothioneins (MTs) are a superfamily of ubiquitously-expressed metal-binding proteins that can be upregulated by exposure to metal, oxidative stress and immune system challenge [47, 48]. MTs are well-known small, cystein-rich proteins that have the ability to bind with high affinity to heavy metal ions, and whose synthesis is regulated by concentrations of metals [49]. MTs play roles in the metabolism of non-toxic essential metals (zinc and copper), as well as toxic heavy metals (cadmium, mercury, etc) [50]. Species of the genus *Crassostrea* have been used in ecotoxicological bioassays to assess the bioaccumulation of Cd and Zn in field conditions [51] and in transplant assays [52, 53]. When comparing the genome of *C. angulata* with that of *C. gigas*, the GO terms (which describe molecular function) associated with binding to ions of zinc and other metals present in *C. angulata* account for the largest number of polymorphic genes (Single nucleotide polymorphisms: SNP) observed [37]. In that context the location of MT genes in *Crassostrea gigas/angulata* genome represents an important advance in the study of those genes in relation to chromosome mapping.

In this work we have assembled BAC sequences available in the GeneBank database of the *C. gigas* oyster and obtained BAC-contig sequences longer than BAC sequences. This approach has allowed us to annotate larger regions than can be done at the level of BACs, and provides knowledge of larger regions of the genome. After annotation we have elucidated the structure of BAC-contigs and, after comparison with the scaffolds of the *C. gigas* genome database, a large number of inconsistencies have been revealed. In many cases, several genes belonging to the same BAC-contigs were mapped in partial regions of different scaffolds in the *C. gigas* database. The main consequence is that a substantial fraction of the genome scaffolds reported by Zhang et al. [40] are incorrectly assembled. These results are in line with those previously published by Hedgecock et al. [38], who after making second-generation linkage maps, with SNP markers, for the Pacific oyster *C. gigas*, observed that more than 38% of the scaffolds with two or more SNPs contained SNPs that unexpectedly map to two or more linkage groups. In our study it is observed that the more genes per BAC-contig, the more scaffolds to which contigs map. Thus, the higher the level of assembly, the greater the number of mistakes (i.e. mis-assemblies) that are made. In *C. gigas*, it has been reported previously that classifying scaffolds by the number of SNPs they contain and by the number of linkage groups to which they map reveals a highly significant, positive association between the two factors. Our data reinforce previous results that claim that misassembled scaffolds comprise large blocks, with the implication that, if the ordering of contigs and genes within scaffolds cannot always be accurate, results about the clustering of gene families, like *Hox* genes [54], should be regarded as provisional until the assembly of genome scaffolds is either confirmed or improved [38]. Currently, the *C. gigas* genome (oyster assembly v9) is assembled to scaffold level and more than 7000 scaffolds are presented [40]. The use of this technique in other species of shellfish and oysters will open a huge potential for direct and fast analysis of genomes in species of high commercial value.

## Conclusions

Molecular markers for 8 out 10 chromosomes have been developed in *Crassostrea angulata/gigas* that allow the differentiation of all chromosomes in the species. An integrated genetic map anchored to the genome has been obtained. Important genes related to resistance to abiotic stress have been mapped.

## Additional files

Additional file 1: **Table S1.** Summary of BAC-contigs statistics. (DOCX 13 kb)

Additional file 2: **Table S2.** Data of BAC-contigs annotations. (XLSX 19 kb)

Additional file 3: **Table S3.** Summary of Gene Ontology term enrichment statistics. (XLSX 2457 kb)

Additional file 4: **Figure S1.** Comparison chart of significant Gene Ontology terms of genes located in BAC-contigs. The arrows indicate the relationship among the GO categories. Colored boxes indicate enriched Gene Ontology terms found in the study. (PDF 2475 kb)

Additional file 5: **Figure S2.** Comparative analysis of genomic structure of BAC-contig and Genomicus browser. (PDF 356 kb)
