# Supplementary material for: A preliminary integrated genetic map distinguishes every chromosome pair and locates essential genes related to abiotic adaptation of Crassostrea angulata/gigas
Source: BMC Genet. 2018 Nov 15;19:104. doi: 10.1186/s12863-018-0689-5 (PMC6238303; doi:10.1186/s12863-018-0689-5)
Supplement: Supplementary file 1 — Table S1. Summary of BAC-contigs statistics. (DOCX 13 kb) [file 12863_2018_689_MOESM1_ESM.docx]

| **Contig** | **Contig Length** | **Average Length/Sequence** | **Total Sequence Length** | **Number of BACs assembled** |
| --- | --- | --- | --- | --- |
| 1 | 271505 | 159513 | 1276108 | 8 |
| 2 | 254895 | 157166 | 314332 | 2 |
| 3 | 161079 | 157845 | 315691 | 2 |
| 4 | 222359 | 133272 | 666364 | 5 |
| 5 | 204747 | 136755 | 547022 | 4 |
| 6 | 141754 | 126302 | 378906 | 3 |
| 7 | 237501 | 132499 | 397499 | 3 |
| 8 | 238060 | 140963 | 281927 | 2 |
| 9 | 198436 | 115485 | 346456 | 3 |
| 10 | 195233 | 126730 | 253460 | 2 |
| 11 | 277695 | 140160 | 280321 | 2 |
| Average | 218478,55 | 138790,00 | 459826,00 | 3,27 |
| **TOTAL** | **2403264** |  |  | **36** |
