# Supplementary material for: A preliminary integrated genetic map distinguishes every chromosome pair and locates essential genes related to abiotic adaptation of Crassostrea angulata/gigas
Source: BMC Genet. 2018 Nov 15;19:104. doi: 10.1186/s12863-018-0689-5 (PMC6238303; doi:10.1186/s12863-018-0689-5)

GO term:comparison chart BAC-contig 1

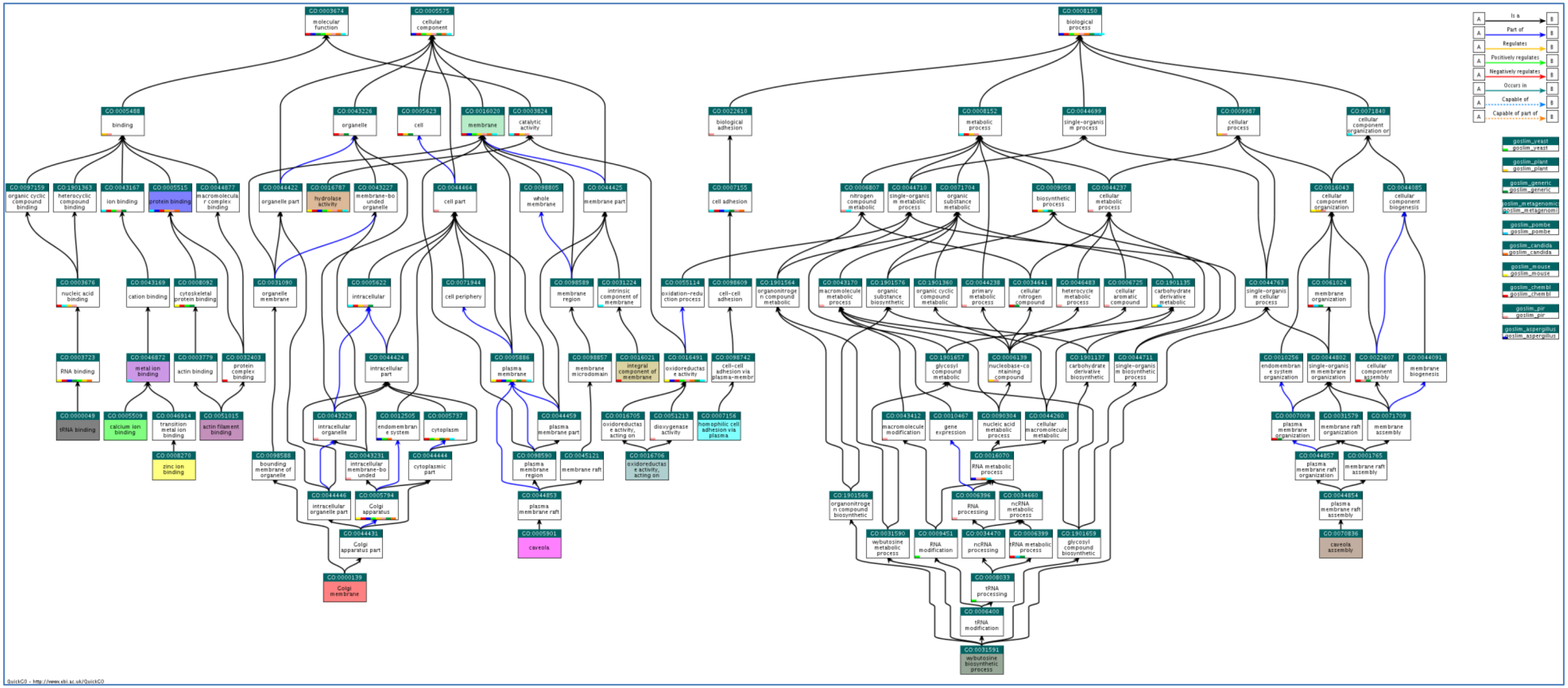

GO term:comparison chart BAC-contig 2

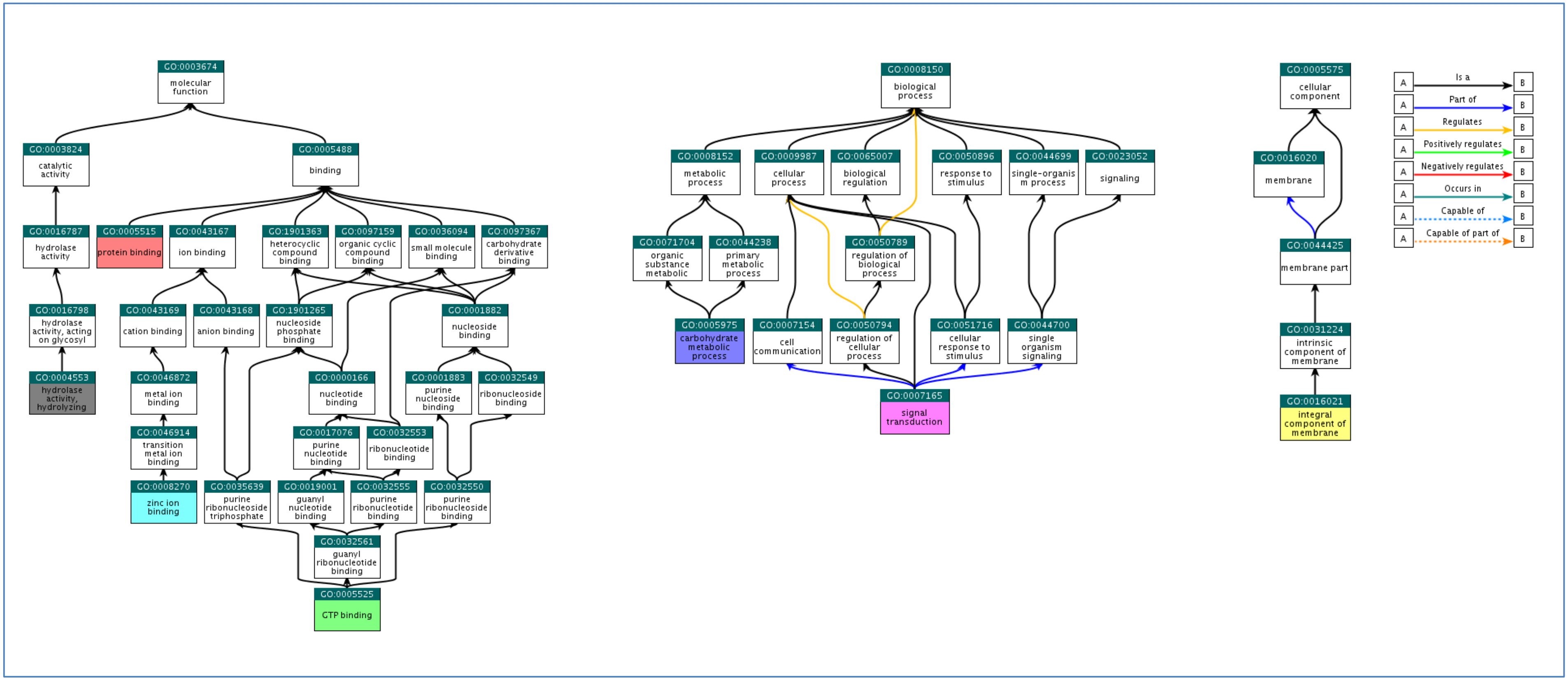

GO term:comparison chart BAC-contig 3

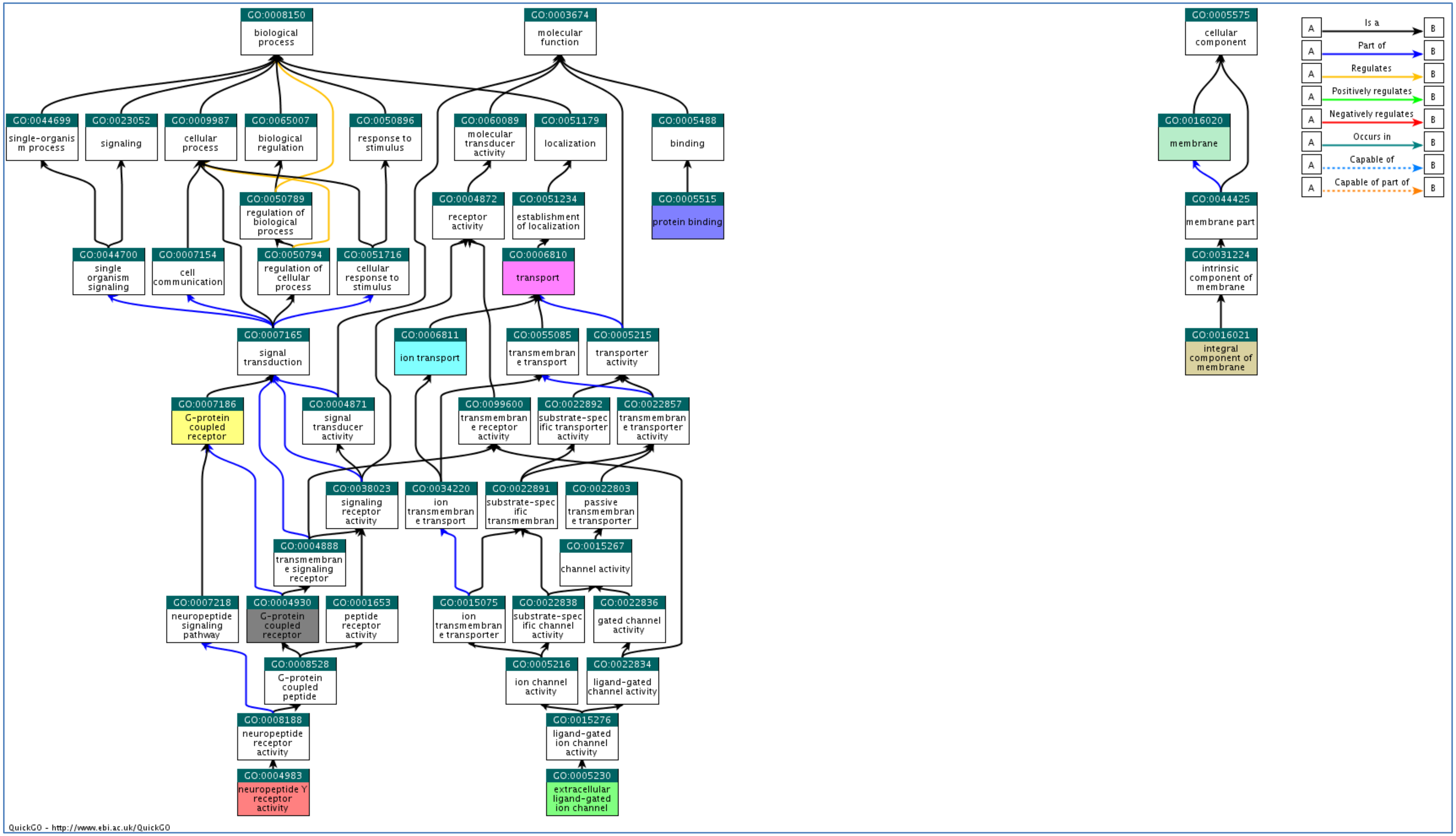

GO term:comparison chart BAC-contig 4

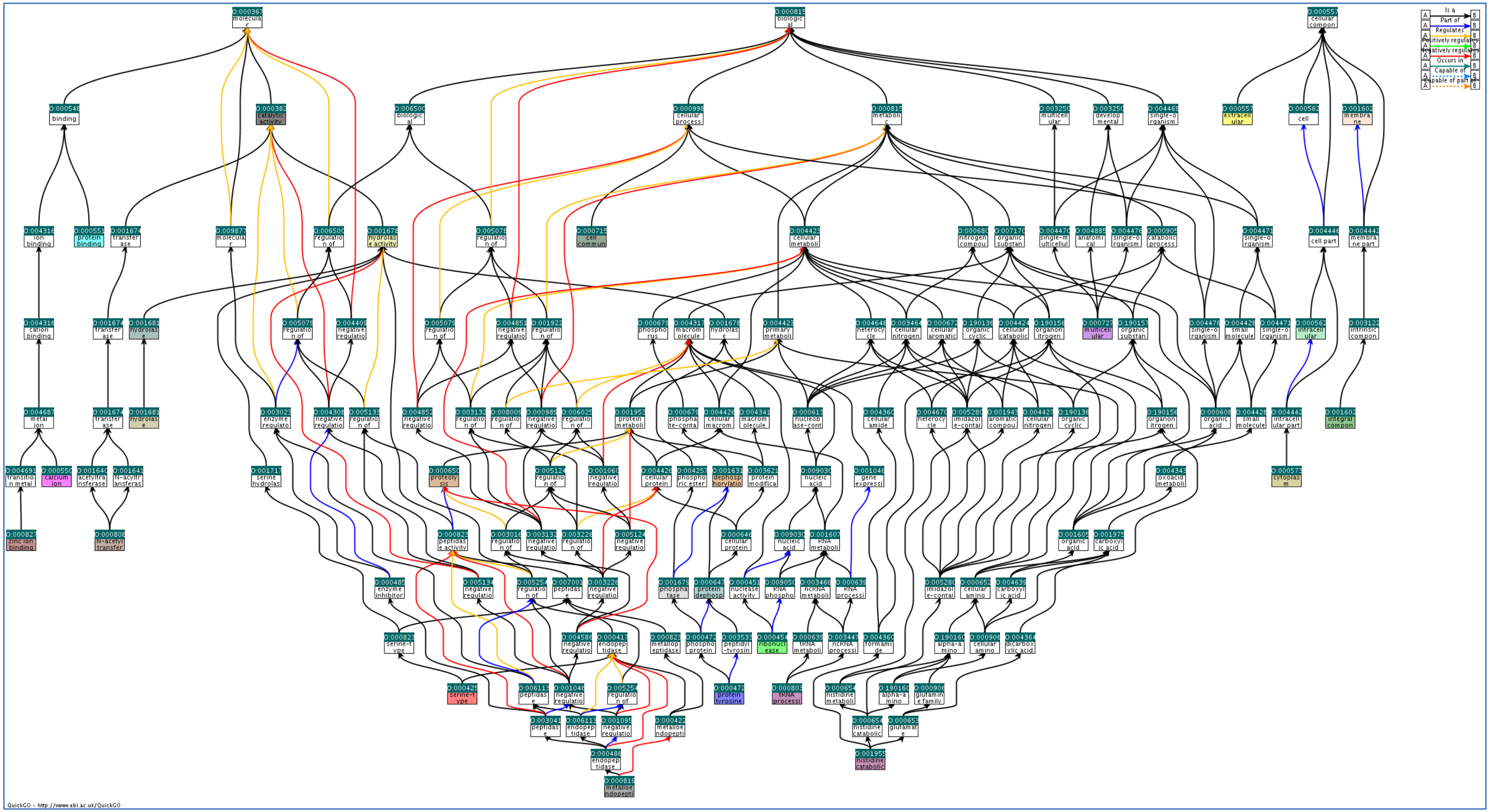

GO term:comparison chart BAC-contig 5

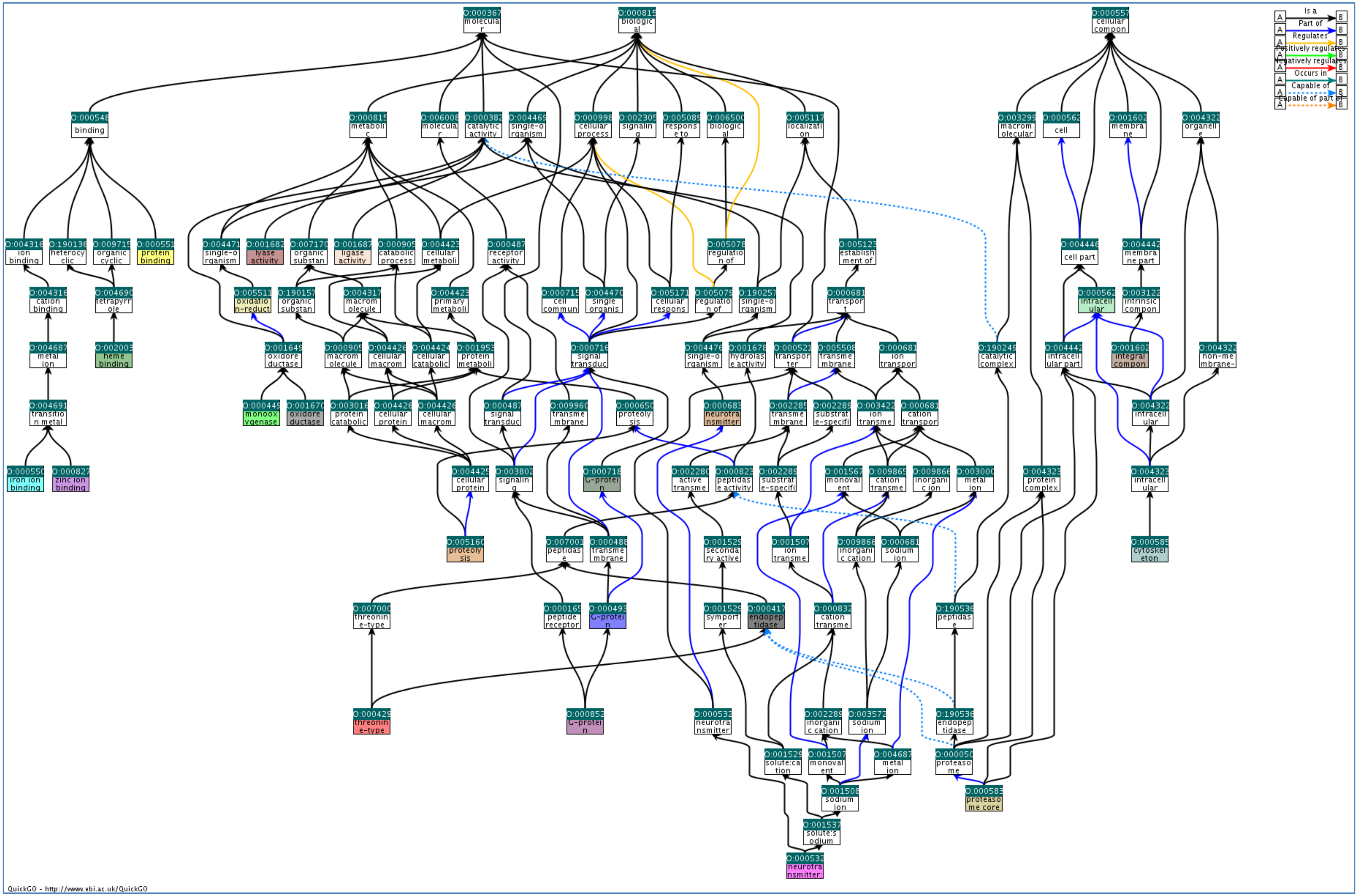

GO term:comparison chart BAC-contig 6

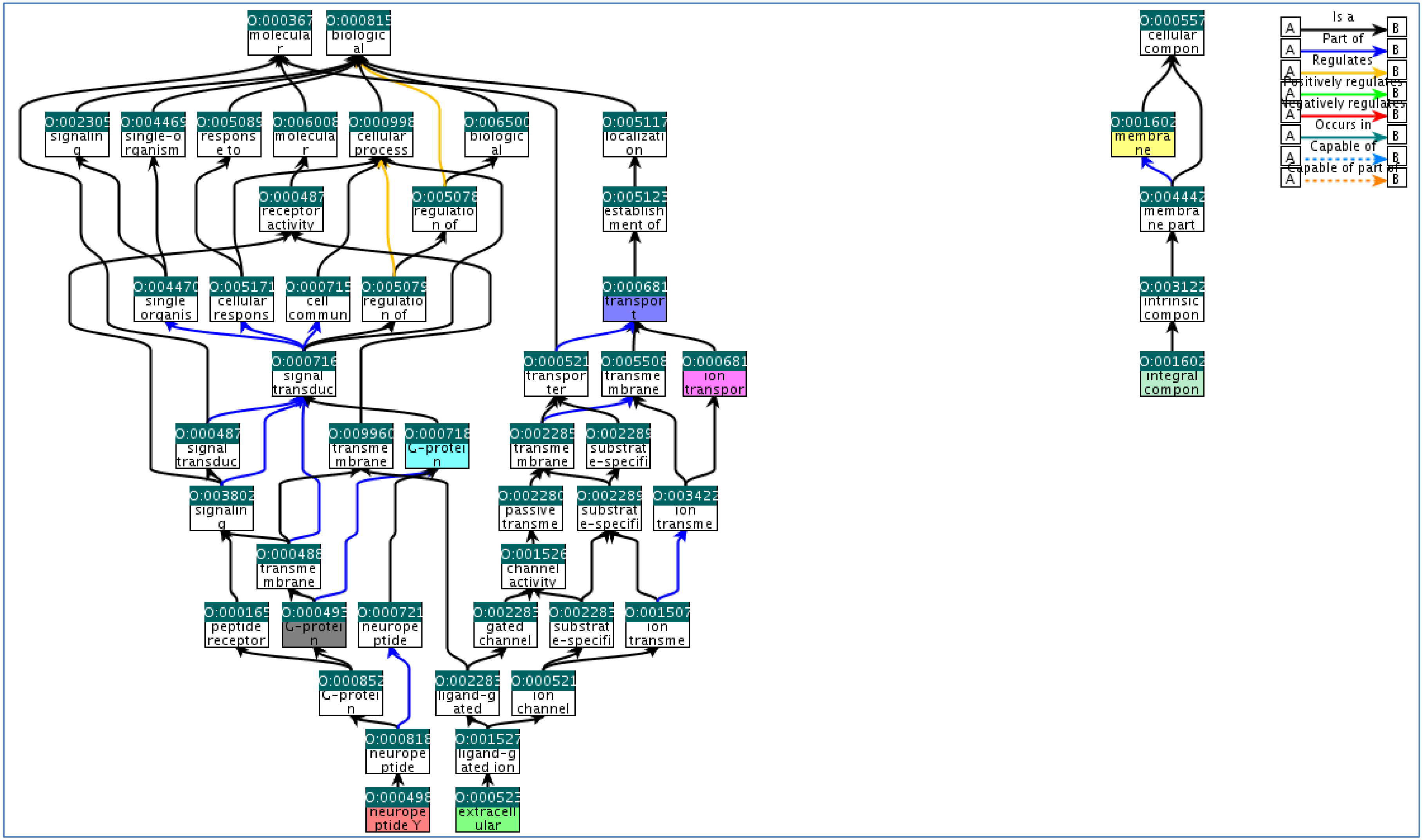

GO term:comparison chart BAC-contig 7

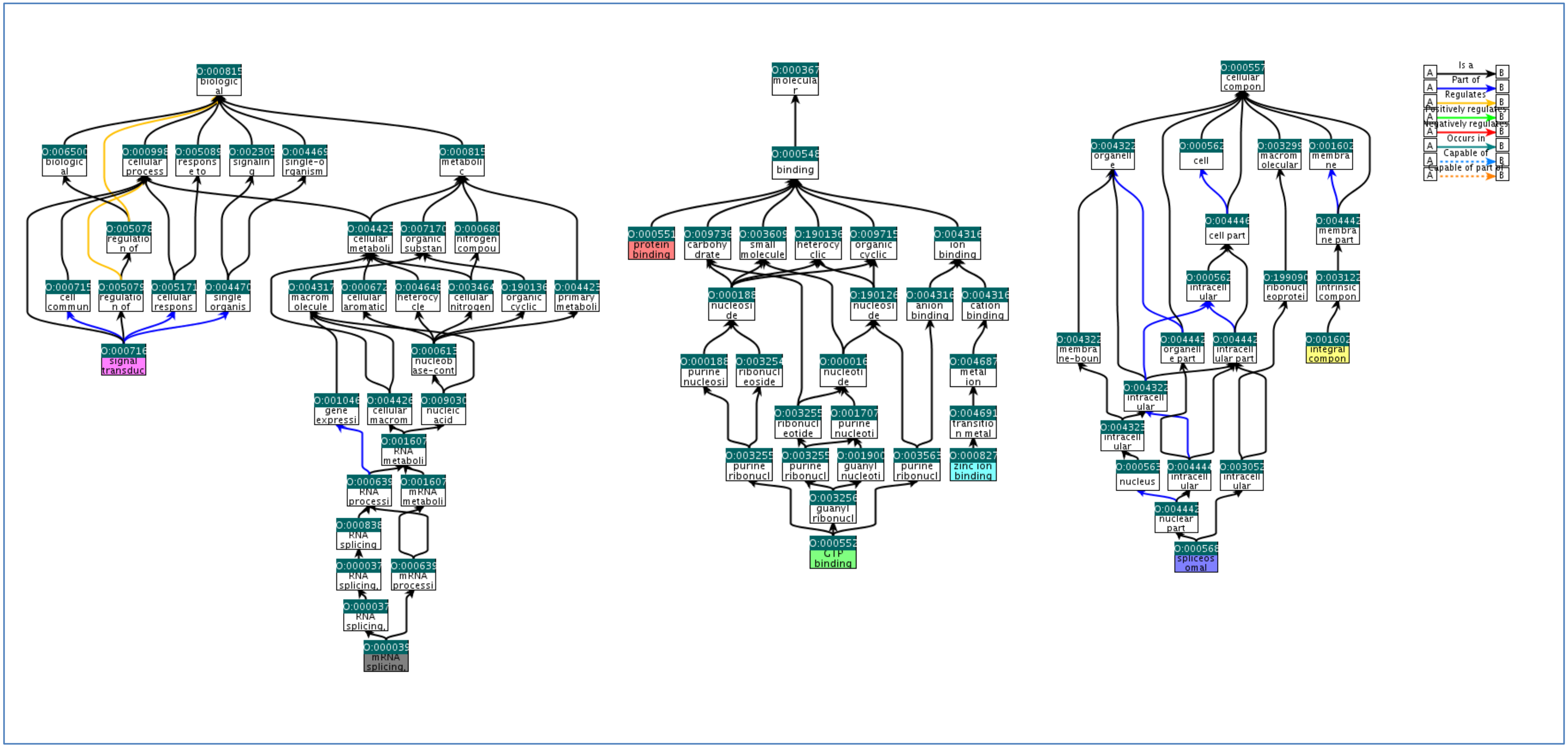

GO term:comparison chart BAC-contig 8

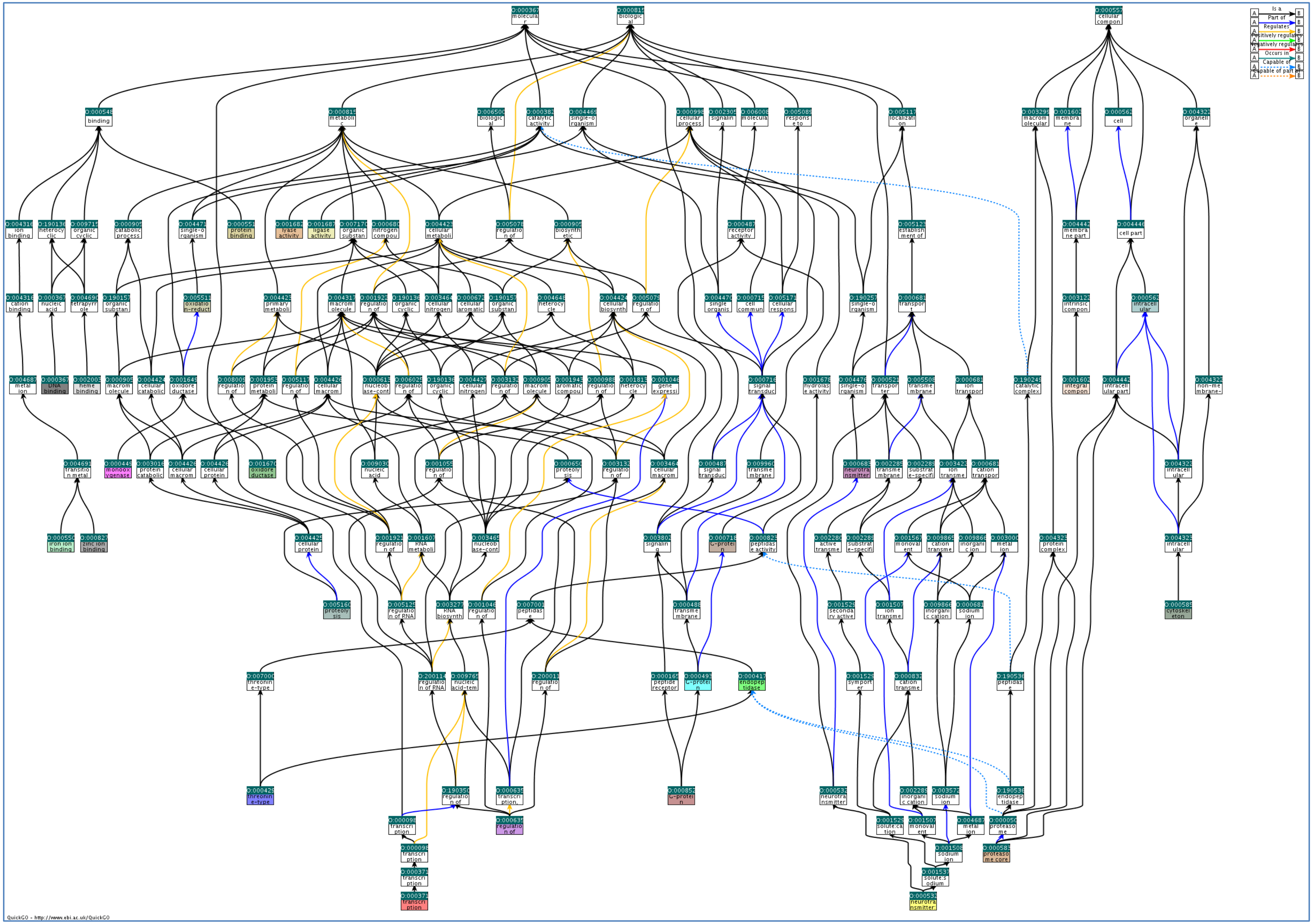

GO term:comparison chart BAC-contig 9

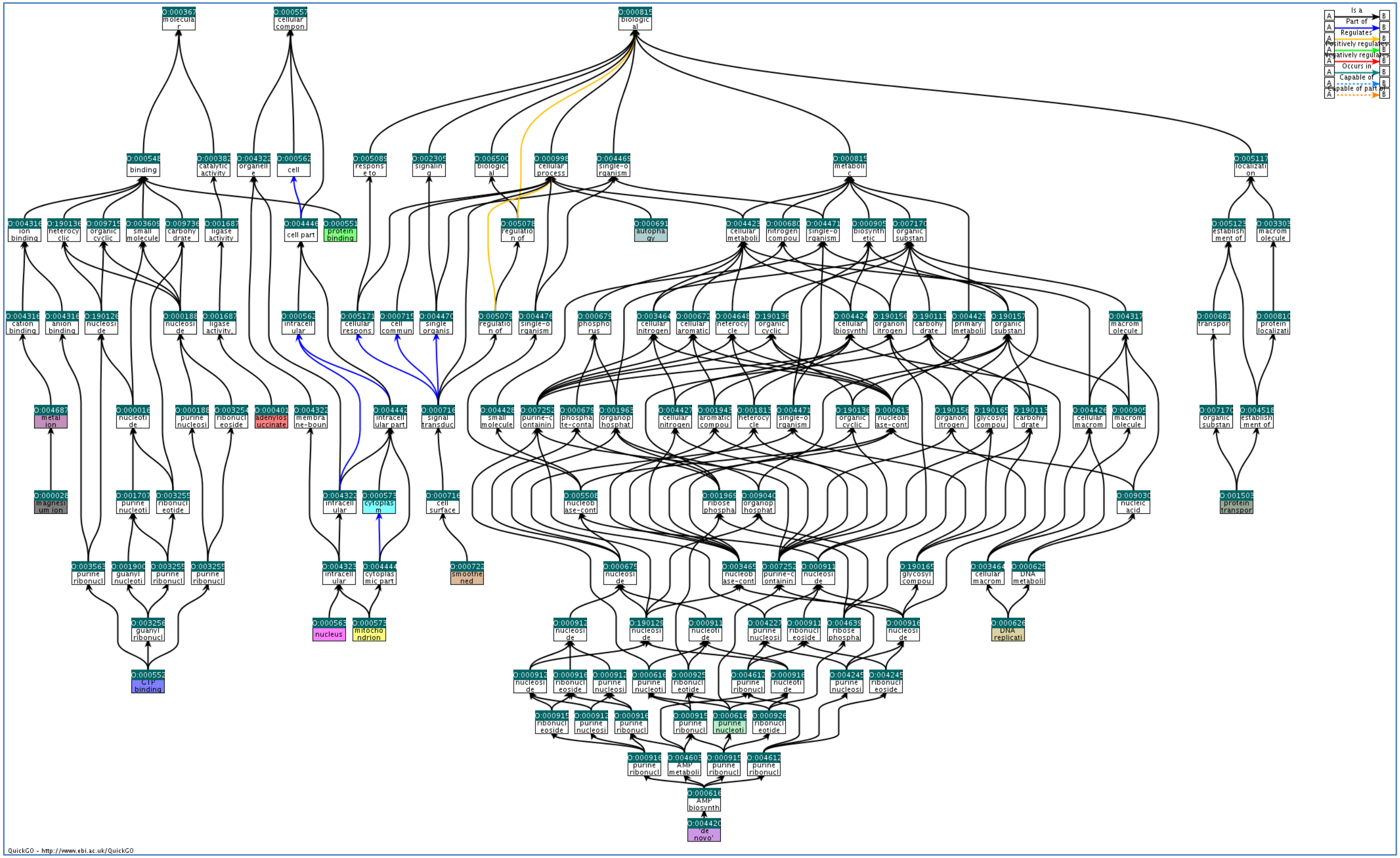

GO term:comparison chart BAC-contig 10

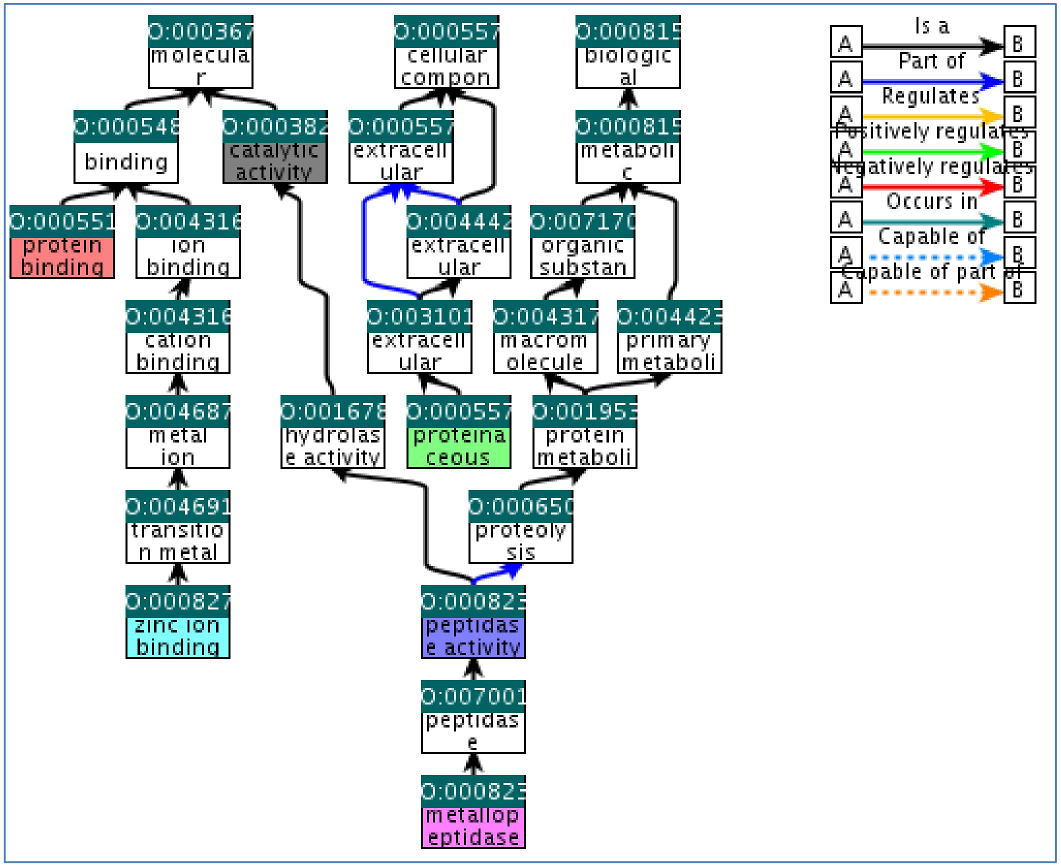

GO term:comparison chart BAC-contig 11

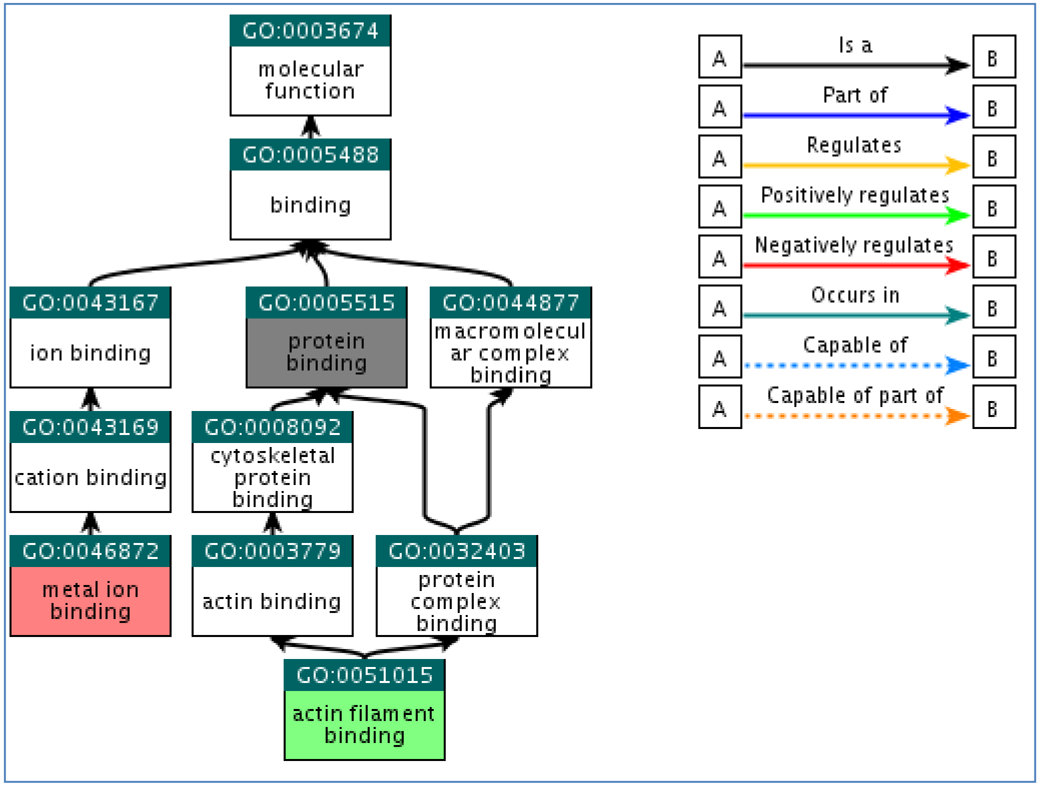

GO term:comparison chart BAC127F5

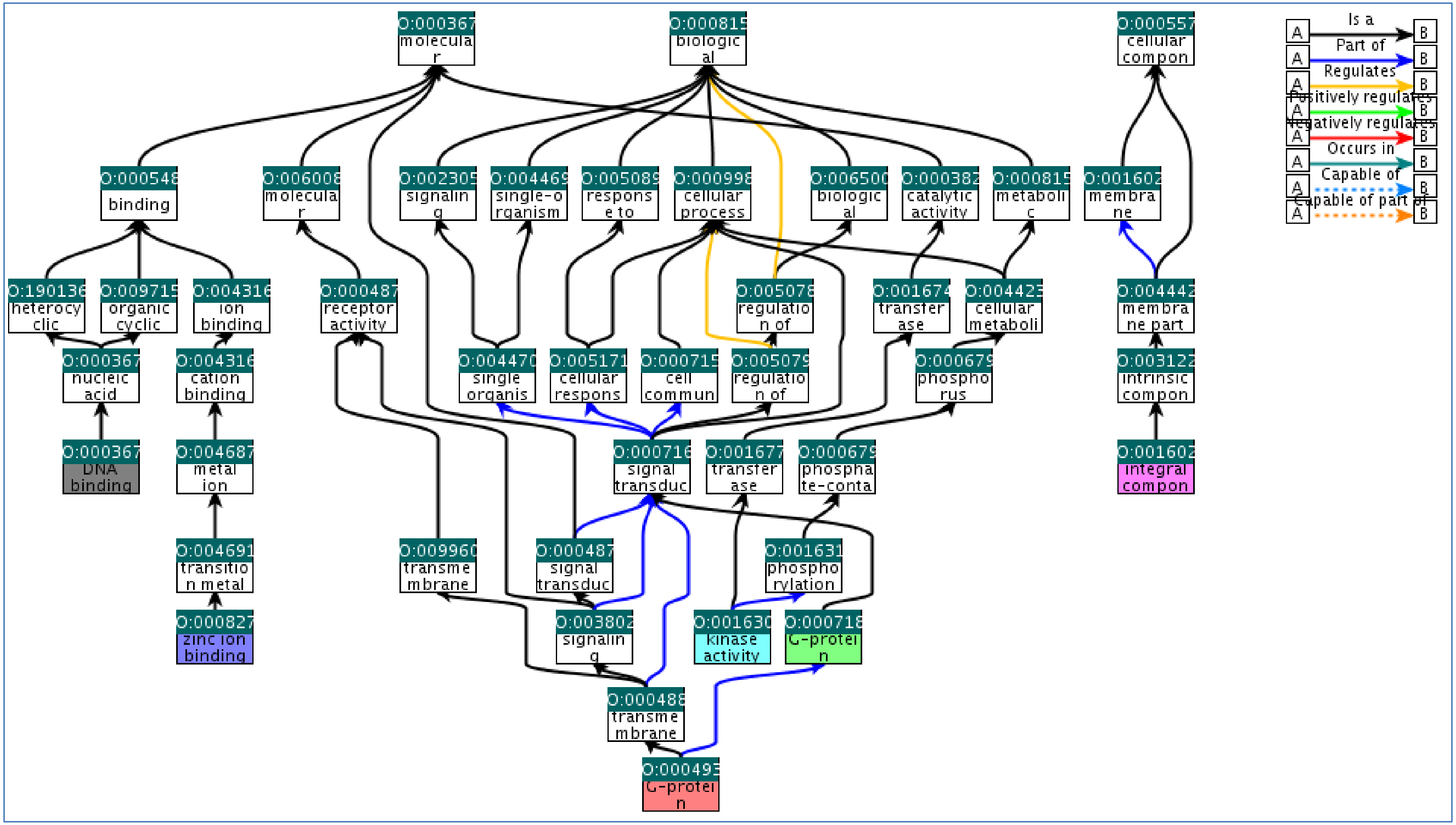

Supplement: Supplementary file 4 — Figure S1. Comparison chart of significant Gene Ontology terms of genes located in BAC-contigs. The arrows indicate the relationship among the GO categories. Colored boxes indicate enriched Gene Ontology terms found in the study. (PDF 2475 kb) [file 12863_2018_689_MOESM4_ESM.pdf]
