# Supplementary material for: A preliminary integrated genetic map distinguishes every chromosome pair and locates essential genes related to abiotic adaptation of Crassostrea angulata/gigas
Source: BMC Genet. 2018 Nov 15;19:104. doi: 10.1186/s12863-018-0689-5 (PMC6238303; doi:10.1186/s12863-018-0689-5)
Supplement: Supplementary file 5 — Figure S2. Comparative analysis of genomic structure of BAC-contig and Genomicus browser. (PDF 356 kb) [file 12863_2018_689_MOESM5_ESM.pdf]

Contig 1

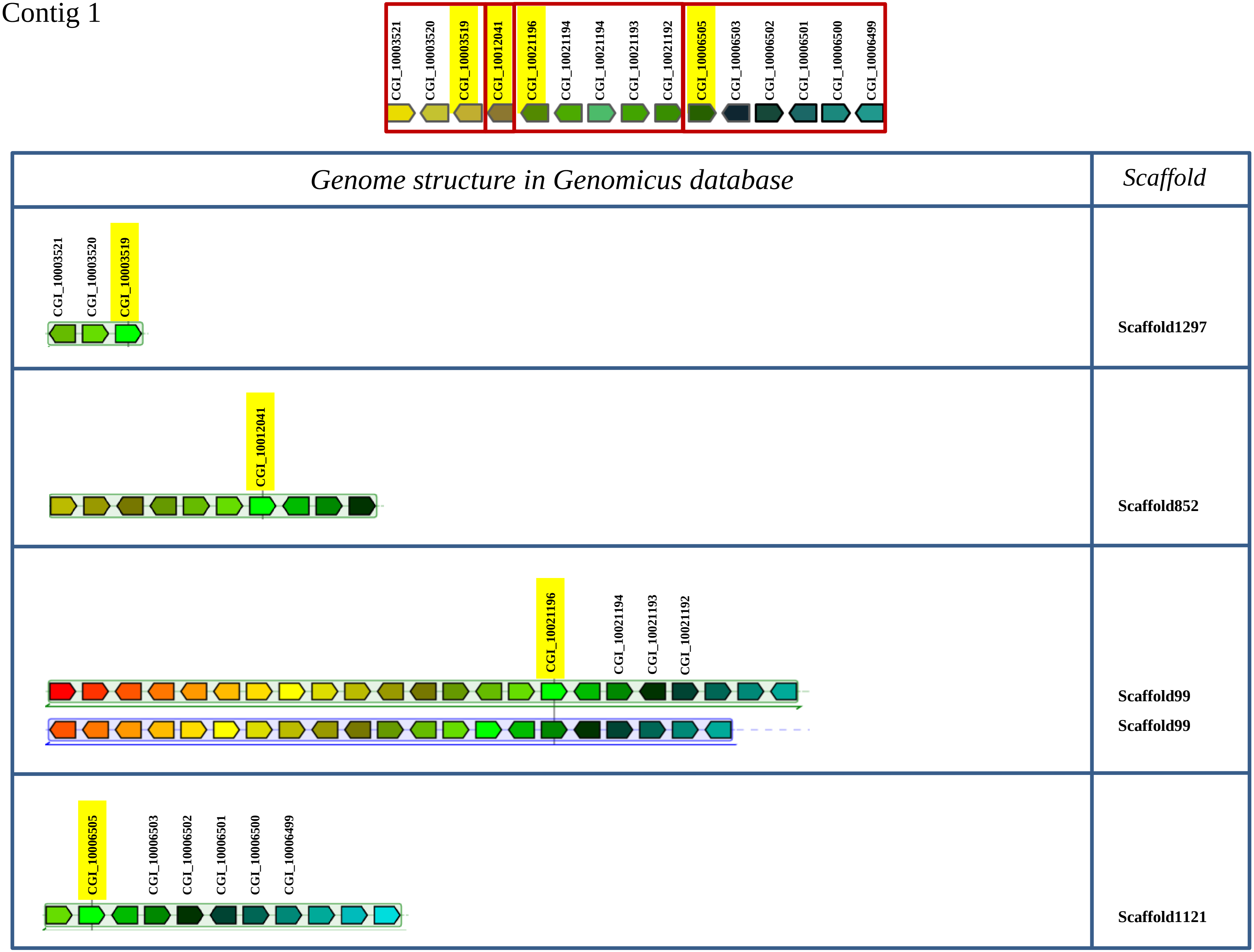
CGI_10003521
CGI_10003520
CGI_10003519
CGI_10012041
CGI_10021196
CGI_10021194
CGI_10021194
CGI_10021193
CGI_10021192
CGI_10006505
CGI_10006503
CGI_10006502
CGI_10006501
CGI_10006500
CGI_10006499
Genome structure in Genomicus database
Scaffold
CGI_10003521
CGI_10003520
CGI_10003519
Scaffold1297
CGI_10012041
Scaffold852
CGI_10021196
CGI_10021194
CGI_10021193
CGI_10021192
Scaffold99
Scaffold99
CGI_10006505
CGI_10006503
CGI_10006502
CGI_10006501
CGI_10006500
CGI_10006499
Scaffold1121

Contig 2

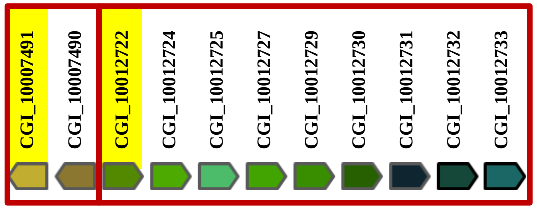

Genome structure in Genomicus database	*Scaffold*
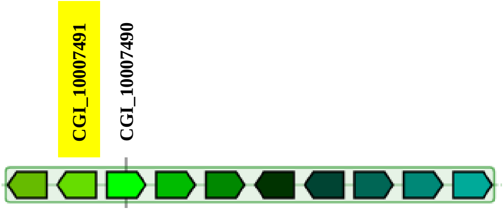	**Scaffold870**
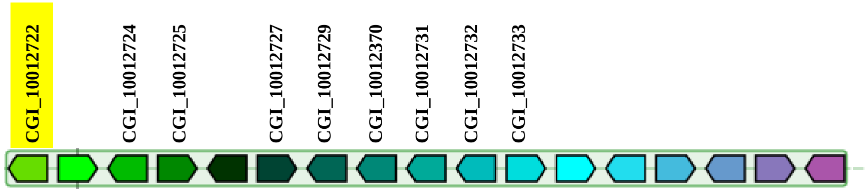	**Scaffold617**

Contig 3

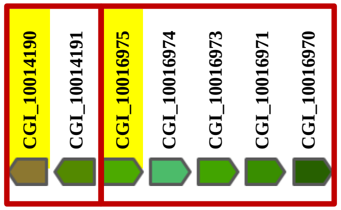

Genome structure in Genomicus database	*Scaffold*
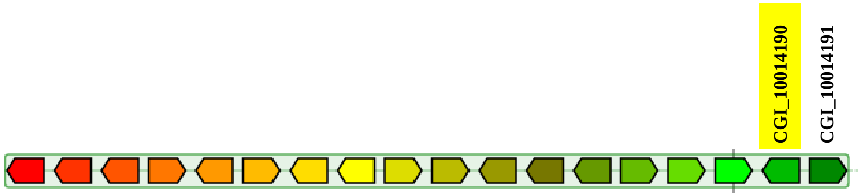	**Scaffold631**
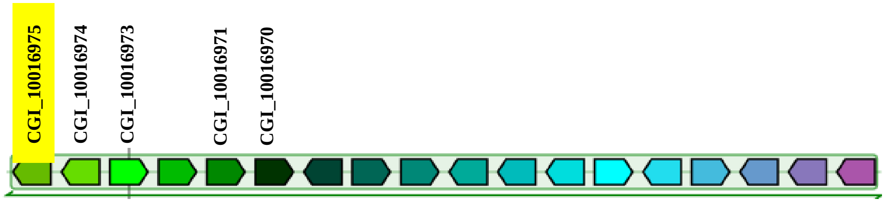	**Scaffold117**

Contig 4

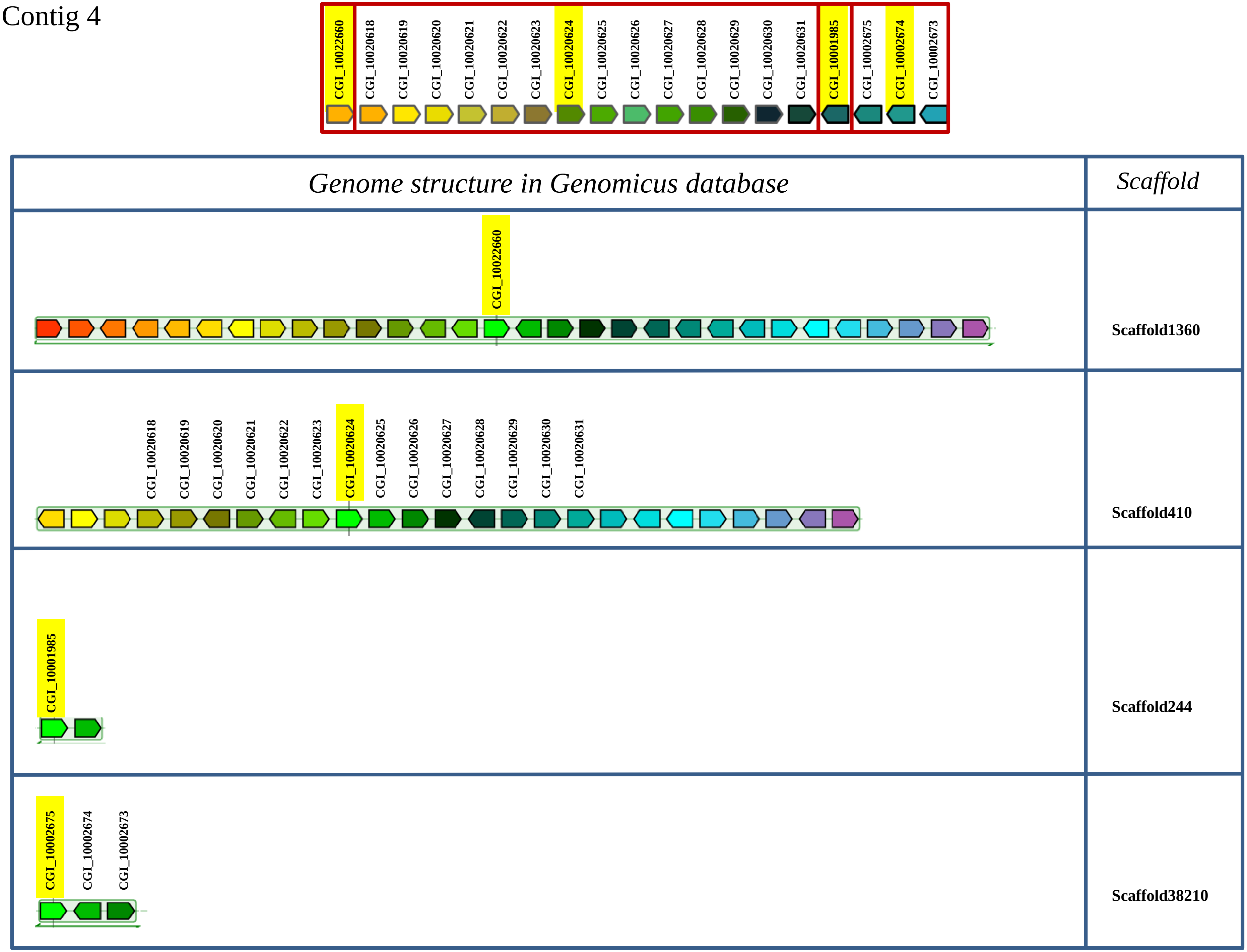
CGI_10022660
CGI_10020618
CGI_10020619
CGI_10020620
CGI_10020621
CGI_10020622
CGI_10020623
CGI_10020624
CGI_10020625
CGI_10020626
CGI_10020627
CGI_10020628
CGI_10020629
CGI_10020630
CGI_10020631
CGI_10001985
CGI_10002675
CGI_10002674
CGI_10002673
Genome structure in Genomicus database
Scaffold
CGI_10022660
Scaffold1360
CGI_10020618
CGI_10020619
CGI_10020620
CGI_10020621
CGI_10020622
CGI_10020623
CGI_10020624
CGI_10020625
CGI_10020626
CGI_10020627
CGI_10020628
CGI_10020629
CGI_10020630
CGI_10020631
Scaffold410
CGI_10001985
Scaffold244
CGI_10002675
CGI_10002674
CGI_10002673
Scaffold38210

Contig 5

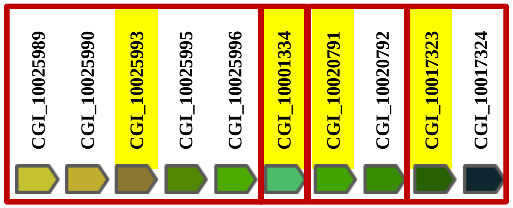
CGI_10025989
CGI_10025990
CGI_10025993
CGI_10025995
CGI_10025996
CGI_10001334
CGI_10020791
CGI_10020792
CGI_10017323
CGI_10017324

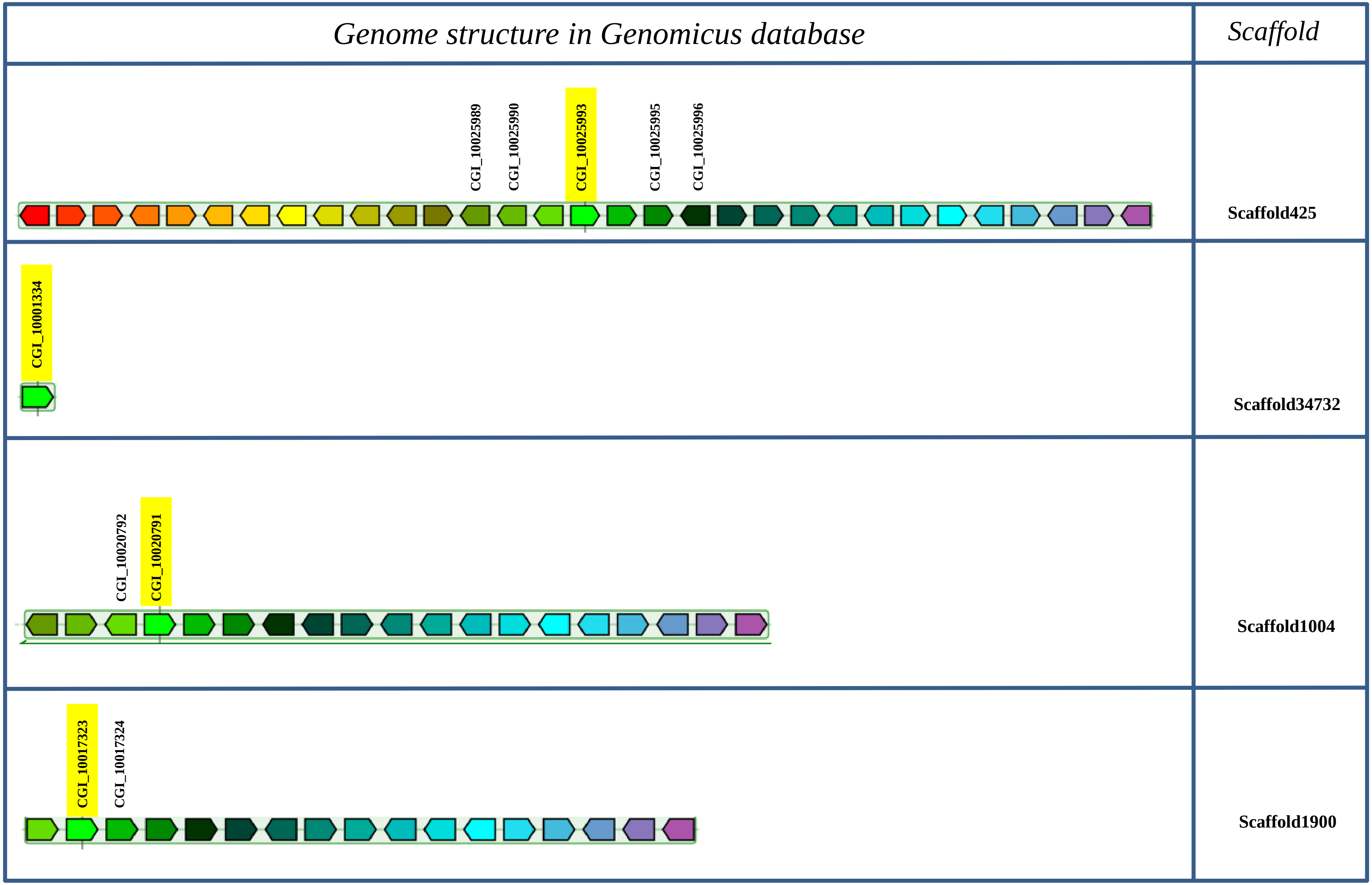
Genome structure in Genomicus database
Scaffold
CGI_10025989
CGI_10025990
CGI_10025993
CGI_10025995
CGI_10025996
Scaffold425
CGI_10001334
Scaffold34732
CGI_10020792
CGI_10020791
Scaffold1004
CGI_10017323
CGI_10017324
Scaffold1900

Contig 6

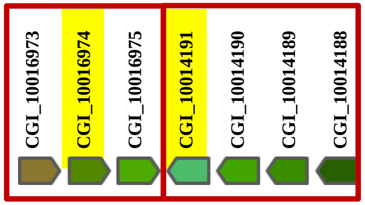

Genome structure in Genomicus database	*Scaffold*
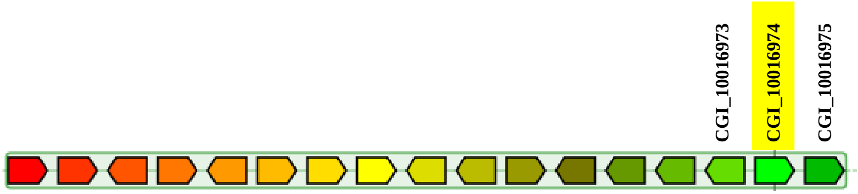	**Scaffold117**
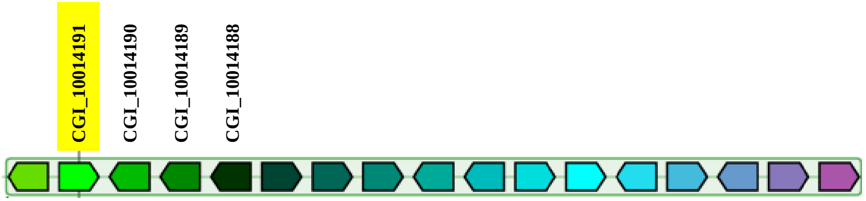	**Scaffold631**

Contig 7

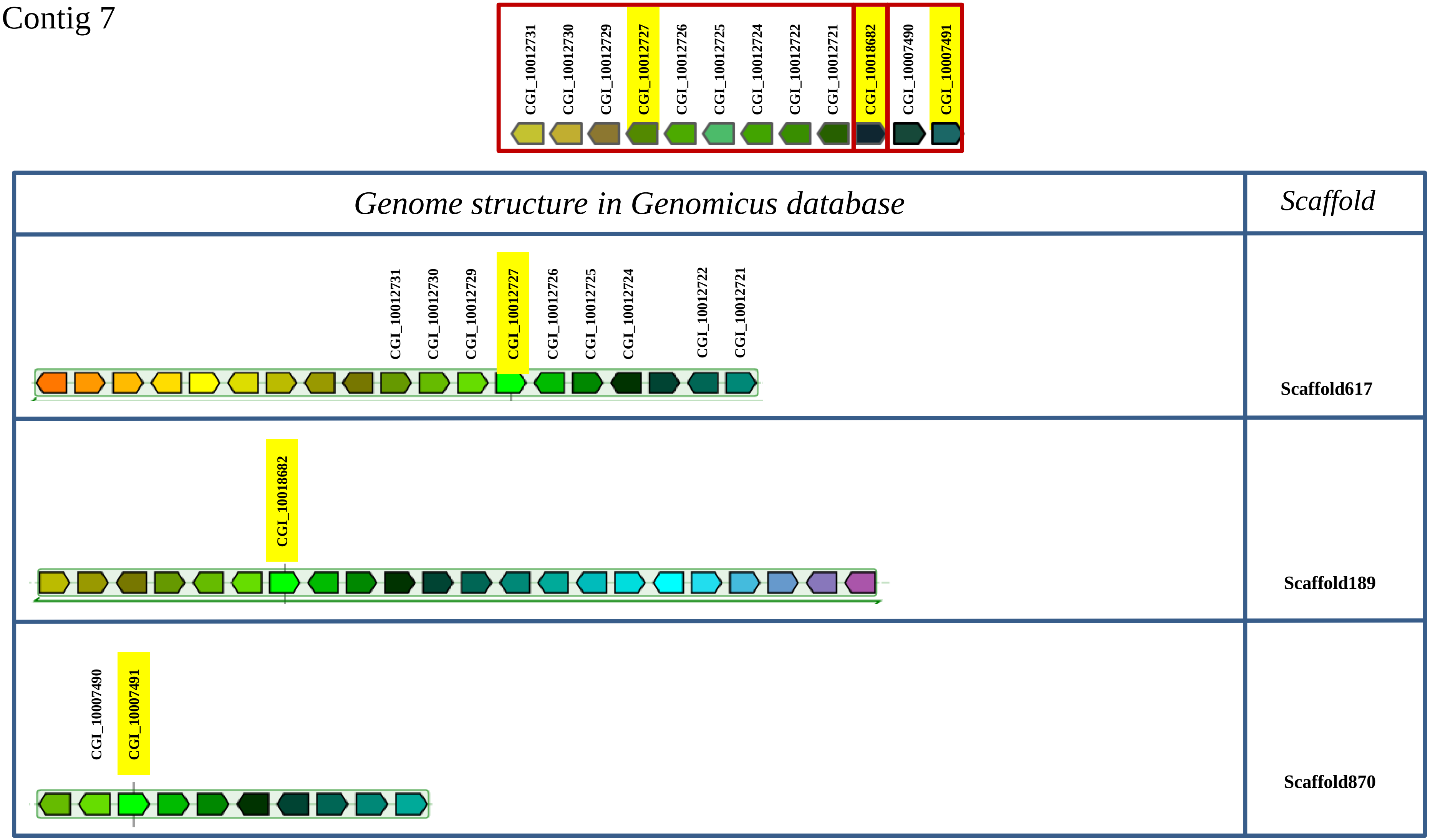
CGI_10012731
CGI_10012730
CGI_10012729
CGI_10012727
CGI_10012726
CGI_10012725
CGI_10012724
CGI_10012722
CGI_10012721
CGI_10018682
CGI_10007490
CGI_10007491
Genome structure in Genomicus database
Scaffold
CGI_10012731
CGI_10012730
CGI_10012729
CGI_10012727
CGI_10012726
CGI_10012725
CGI_10012724
CGI_10012722
CGI_10012721
Scaffold617
CGI_10018682
Scaffold189
CGI_10007490
CGI_10007491
Scaffold870

Contig 8

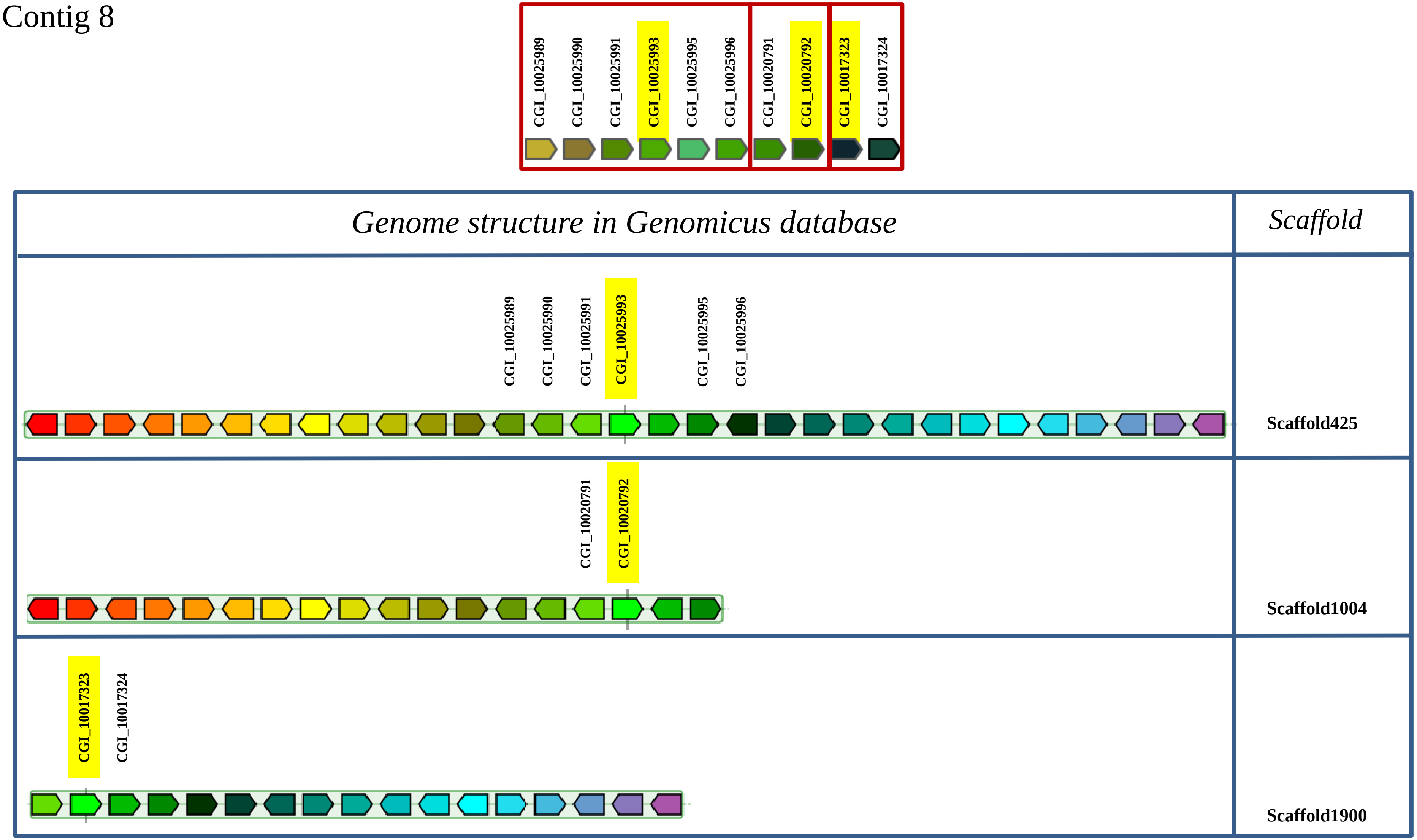

CGI_10025989
CGI_10025990
CGI_10025991
CGI_10025993
CGI_10025995
CGI_10025996
CGI_10020791
CGI_10020792
CGI_10017323
CGI_10017324
Genome structure in Genomicus database
Scaffold
CGI_10025989
CGI_10025990
CGI_10025991
CGI_10025993
CGI_10025995
CGI_10025996
Scaffold425
CGI_10020791
CGI_10020792
Scaffold1004
CGI_10017323
CGI_10017324
Scaffold1900

Contig 9

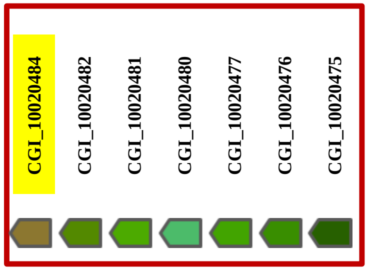

Genome structure in Genomicus database	*Scaffold*
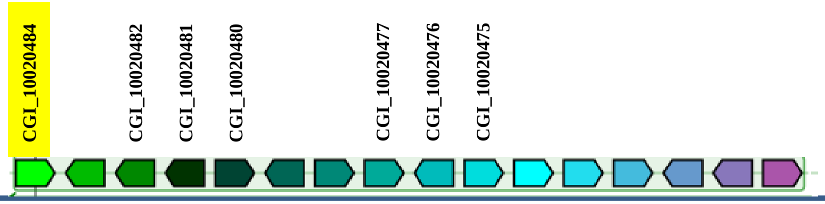	**Scaffold388**

Contig 10

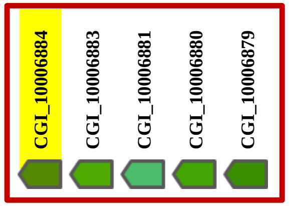

Genome structure in Genomicus database	*Scaffold*
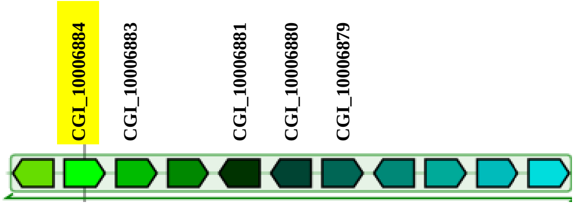	**Scaffold2448**

Contig 11

##gff-version
XM_011457369
AY331706

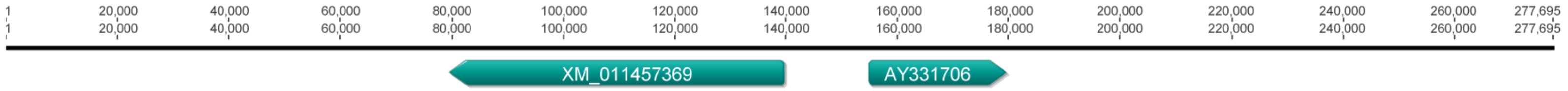

This BAC contians two genes (XM_011457369 and AY331706) not described in Genomicus database. Genes were annotated using BLASTn. Figure has being made with program Geneious.

BAC127F5

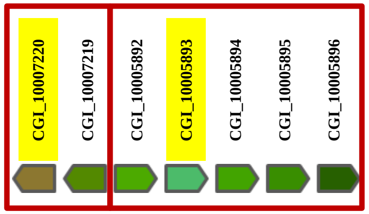

Genome structure in Genomicus database	*Scaffold*
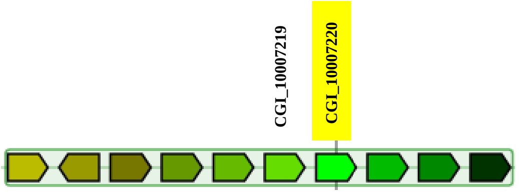	**Scaffold42558**
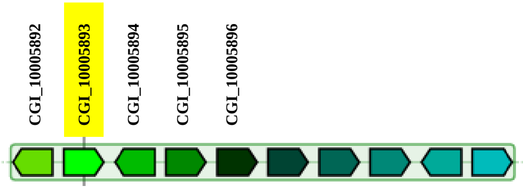	**Scaffold41988**
